# CLAE: A High‐Fidelity Nanopore Sequencing Strategy for Read‐Level Viral Variant Detection and Environmental RNA Virus Discovery

**DOI:** 10.1002/advs.202505978

**Published:** 2025-09-11

**Authors:** Hannah Yu, Sarah Golconda, Ga‐Eun Lee, Dantong Xue, Guillermo Domínguez‐Huerta, James M. Wainaina, Benjamin Bolduc, Shashanka Murthy, Shihyoung Kim, Seth Faith, Shan‐Lu Liu, Jiyoung Lee, Michael Oglesbee, Matthew B Sullivan, Sanggu Kim

**Affiliations:** ^1^ Center for Retrovirus Research The Ohio State University Columbus OH 43210 USA; ^2^ Department of Veterinary Biosciences The Ohio State University Columbus OH 43210 USA; ^3^ Infectious Diseases Institute The Ohio State University Columbus OH 43210 USA; ^4^ Translational Data Analytics Institute The Ohio State University Columbus OH 43210 USA; ^5^ Department of Computer Science and Engineering The Ohio State University Columbus OH 43210 USA; ^6^ Center of Microbiome Science The Ohio State University Columbus OH 43210 USA; ^7^ Department of Microbiology The Ohio State University Columbus OH 43210 USA; ^8^ Biology Department Woods Hole Oceanographic Institution Woods Hole MA 02543 USA; ^9^ Division of Environmental Health Sciences College of Public Health The Ohio State University Columbus Ohio 43210 USA; ^10^ Department of Food Science & Technology The Ohio State University Columbus Ohio 43210 USA; ^11^ Department of Civil Environmental and Geodetic Engineering The Ohio State University Columbus OH 43210 USA; ^12^ Center for RNA Biology The Ohio State University Columbus OH 43210 USA

**Keywords:** high‐fidelity long‐reads, metaviromics, nanopore sequencing, ocean virome, RNA viruses, rolling circle amplification, SARS‐CoV‐2

## Abstract

High‐fidelity (HF) long‐read sequencing enables accurate profiling of microorganisms and pathogens at single‐molecule resolution. However, current Oxford Nanopore Technologies (ONT)—a revolutionary platform offering real‐time, portable sequencing at relatively low instrumental cost—suffer from severe read‐length bias, limited accuracy (often <Q20), and low throughput. Here, Circular‐ and Linear‐Amplicon‐Mediated Error Correction (CLAE) is introduced, a biochemical and computational approach that addresses these limitations by integrating hairpin ligation, pre‐circling, single‐stranded DNA linearization, and targeted nickase‐based debranching. CLAE significantly enhances rolling‐circle amplification (RCA) efficiency for long DNA templates, markedly improving Nanopore sequencing yield and accuracy. CLAE achieves Q30‐level accuracy in up to 27% of RCA reads, throughput exceeding 800 Mb per 100 pores, and an N50 of ≈15 Kb (bacterial genome). Moreover, its bidirectional subreads and high throughput substantially boost accuracy without compromising read length. CLAE is validated by resolving SARS‐CoV‐2 quasi‐species from community wastewater and recovering novel, full‐length RNA virus genomes from marine samples. CLAE enables precise variant detection in complex samples and corrects short‐read misassemblies, significantly broadening ONT's utility in metaviromics, epidemiology, and environmental surveillance. Thus, CLAE establishes a versatile, field‐compatible platform for high‐fidelity viral genome sequencing in targeted and agnostic contexts.

## Graphical abstract

High‐fidelity Nanopore sequencing offers a cost‐effective, portable path to uncovering viral dark matter in complex environments—but suffers from severe read‐length bias, low throughput, and limited accuracy. This study traces these limitations to core biochemical barriers and introduces CLAE, a foundational high‐fidelity platform that enables accurate, read‐level viral genomics—paving the way for real‐time, field‐deployable metaviromics in wastewater, oceans, and beyond.

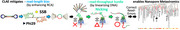

## 1 Introduction

Viruses are among the most diverse and abundant life forms on Earth. RNA viruses, in particular, exhibit extraordinary mutational diversity due to their high mutation rates.^[^ ^1^, ^2^ ^]^ Hundreds have been characterized for their critical roles in human, animal, and plant diseases. However, the devastating impacts of emerging and re‐emerging viruses—including SARS‐CoV‐2— underscore the urgent need for sensitive tools to detect and monitor these pathogens. Beyond human health, RNA viruses also play critical roles in biogeochemical cycles, influencing the ocean's ability to buffer against climate change.^[^ ^3^ ^]^ Recent metaviromic surveys have greatly expanded our understanding of RNA virus diversity, revealing at least five new phyla based on a single conserved marker gene, the RNA‐dependent RNA polymerase (RdRp).^[^ ^4^, ^5^, ^6^ ^]^ It is not surprising that the few hundred RNA viruses studied to date likely represent only a tiny fraction of the estimated 10^31^ viruses on the planet—most with unknown genotypes.^[^ ^7^ ^]^

Efforts to discover and monitor RNA viruses face major obstacles due to the inherent fragility of RNA^[^ ^8^ ^]^ and technical challenges associated with sequencing.^[^ ^9^, ^10^ ^]^ Current detection tools are largely population‐based and optimized for dominant species, making them inefficient for identifying low‐frequency variants in mixed samples. These technical limitations hinder early warning systems such as wastewater surveillance^[^ ^11^, ^12^, ^13^, ^14^ ^]^ and impede the detection of drug‐resistant subspecies that could inform treatment strategies.^[^ ^15^ ^]^ Detecting such variants requires distinguishing subspecies‐level nucleotide differences across individual RNA molecules—something short‐read sequencing struggles to achieve. While molecular barcoding has been explored to improve resolution,^[^ ^16^, ^17^, ^18^, ^19^ ^]^ it is constrained by the short length and technical complexity of synthetic barcodes, which can affect both sensitivity and accuracy.^[^ ^20^, ^21^ ^]^

Pacific Biosciences’ circular consensus sequencing (CCS) has emerged as a powerful alternative. By generating long (10–25 kb), highly accurate (99.8%) reads, PacBio CCS enables single‐molecule resolution of viral populations, uncovering intra‐host diversity in HIV‐1,^[^ ^22^ ^]^ influenza,^[^ ^23^, ^24^ ^]^ Hepatitis C viruses,^[^ ^25^, ^26^ ^]^ SARS‐CoV‐2^[^ ^27^ ^]^ and mycoviruses.^[^ ^28^ ^]^ In addition, PacBio's accurate long reads have been applied to simplified haplotyping,^[^ ^29^ ^]^ transcript isoform profiling,^[^ ^30^ ^]^ complex microbial community analysis,^[^ ^31^, ^32^ ^]^ and metagenomic assembly of DNA viruses^[^ ^33^, ^34^, ^35^, ^36^ ^]^ — applications often impractical with short reads.

Oxford Nanopore Technologies (ONT) offers another long‐read platform that enables portable, real‐time sequencing at significantly lower instrumental cost. However, high‐fidelity sequencing on ONT, which relies on self‐ligation of double‐stranded DNA for rolling circle amplification (RCA),^[^ ^37^, ^38^, ^39^, ^40^ ^]^ suffer from substantial read‐length bias and limited consensus accuracy (frequently <Q20). Self‐ligation efficiency drops more than tenfold for DNA fragments >2 kb versus shorter ones (300–500 nt), likely due to differential folding properties and entropic penalties.^[^ ^41^, ^42^, ^43^ ^]^ These methods also show five to tenfold lower read accuracy than PacBio CCS,^[^ ^37^, ^38^, ^39^, ^40^, ^44^ ^]^ attributed to ONT's strand‐specific k‐mer errors^[^ ^45^, ^46^ ^]^ and difficulty generating long RCA reads. While approaches like R2C2+UMI improve accuracy,^[^ ^47^ ^]^ they do not resolve the intrinsic read‐length bias of self‐ligation‐based RCA. Hairpin‐ligation‐mediated RCA, used in PacBio CCS^[^ ^48^ ^]^ and MrHAMER,^[^ ^40^ ^]^ avoids the need for DNA bending and can reduce length bias. However, when adapted for ONT, our data show that even hairpin‐based RCA exhibits significant bias, largely due to inefficient amplification of long DNA fragments—which ultimately limits both accuracy and throughput.

All existing HF Nanopore sequencing approaches share two critical limitations: i) a read‐length bias that disproportionately limits coverage of longer and/or structured templates, and ii) low sequencing throughput, likely due to inefficient translocation of structured RCA‐amplified DNA through nanopores (see Table S1, Supporting Information for summary). These challenges represent significant hurdles to routine laboratory adoption and field applications in metaviromics or environmental surveillance.

To directly address these barriers, we developed CLAE (Circular‐ and Linear‐Amplicon‐Mediated Error‐Correction), a high‐fidelity long‐read sequencing method optimized for ONT platforms. CLAE combines biochemical innovations—including hairpin ligation, single‐stranded binding protein stabilization, and targeted nicking—with computational strategies to maximize consensus correction.

We benchmarked CLAE using model DNA and SARS‐CoV‐2 RNA from nasal swabs and wastewater, then applied it to ocean viromes for agnostic discovery of RNA viruses. This approach enabled full‐length reconstruction of novel viral genomes—including correction of a misassembled picorna‐like virus—and improved detection of cryptic variants in local wastewater. Together, our results demonstrate that CLAE enables high‐accuracy, long‐read sequencing of RNA viruses in both clinical and environmental settings, advancing real‐time viral surveillance and discovery.

## 2 Results

### 2.1 Hairpin‐Mediated RCA Exhibits Length Bias

**Figure 1:**
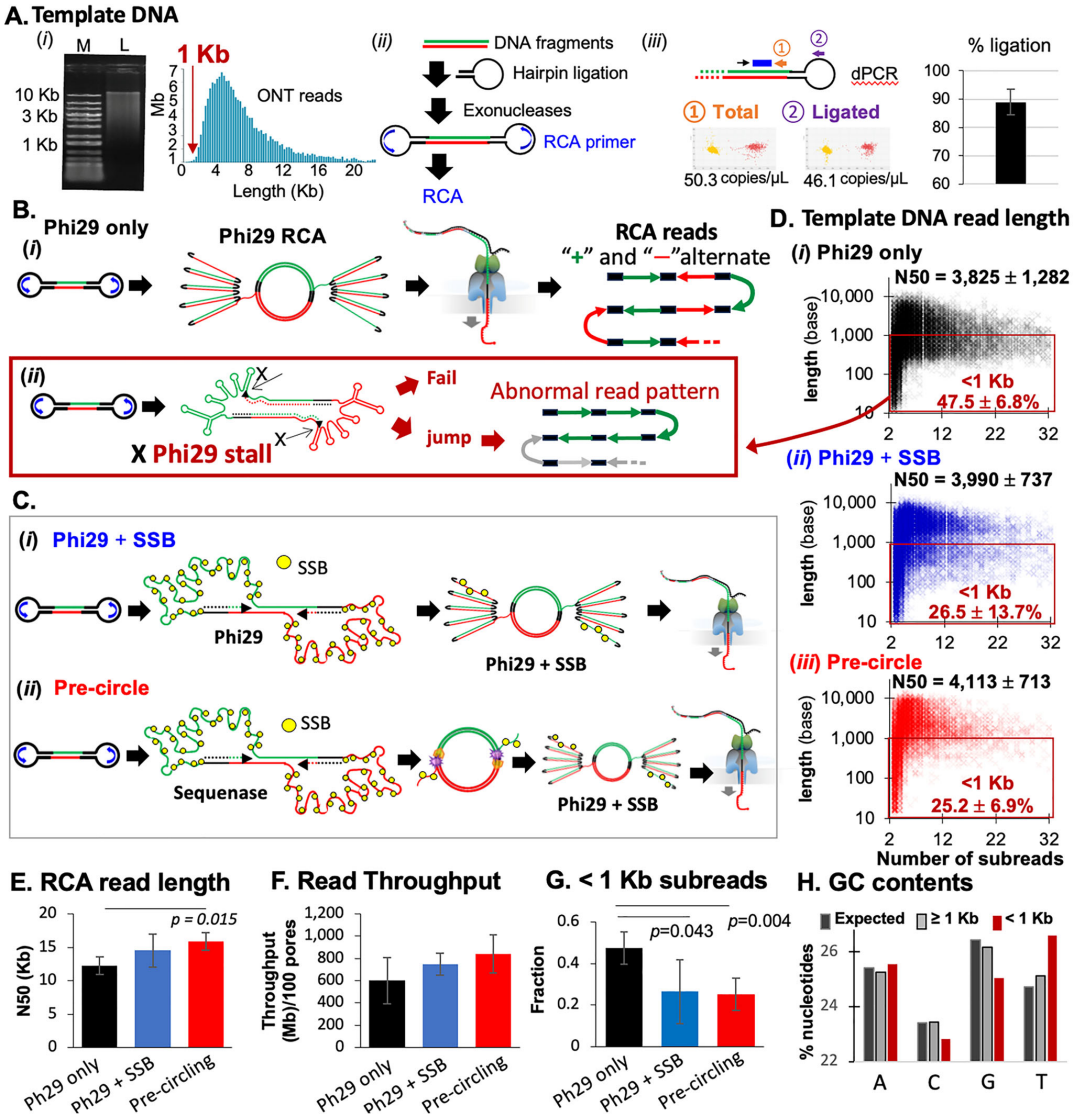
SSB and pre‐circling reduce read‐length bias in RCA‐mediated consensus sequencing. A) Randomly fragmented template DNAs (*i*) were subjected to hairpin ligation and RCA (*ii*). Ligation efficiency was measured by digital PCR (*iii*). B,C) Schematic of RCA amplification using Phi29 alone (B‐*i‐*,‐*ii‐*), Phi29 + SSB (C‐*i‐*) and pre‐circling prior to RCA (C‐*ii‐*). RCA recombination can occur due to Phi29 stall at secondary structures (B‐*ii*‐, arrows). Longer DNA templates are more prone to this stalling, which potentially causes RCA recombination with abnormal read patterns of smaller subreads. D) Length distributions of subreads (y‐axis) are shown for Phi29 only (D‐*i‐, n* = 4), Phi29+SSB (D‐*ii‐, n* = 5), and pre‐circle (D‐*iii‐, n* = 5) conditions. The number of subreads in each RCA read is shown on the x‐axis. N50s are shown on top of the plot. RCA reads exhibiting <1 Kb consensus reads are shown in red boxes (the fractions are denoted by red texts). E–G) Comparison of RCA read length (E), throughput (F), and the proportion of <1Kb consensus reads (G). *p‐*values are from Welch's *t*‐test. H) Nucleotide compositions for ≥1 and <1 Kb reads were compared.

To circumvent the read‐length bias associated with self‐circularization‐mediated RCA addressed above,^[^ ^41^, ^42^, ^43^ ^]^ we employed a hairpin‐ligation‐based RCA, similar to MrHAMER^[^ ^40^ ^]^ and PacBio's SMRT‐bell.^[^ ^48^ ^]^ Randomly fragmented lambda phage DNA or *Escherichia coli* DNA (average 6 Kb fragments, **Figure** **1**A‐*i*‐) served as RCA templates. The hairpin ligation efficiency—a critical determinant of overall sequencing sensitivity—was approximately 89 ± 4% (Figure 1A‐*ii‐ & ‐iii*‐). After exonuclease treatment to remove unligated or partially ligated DNA, RCA was initiated using hairpin‐specific primers and phi29 DNA polymerase.

**Figure 2:**
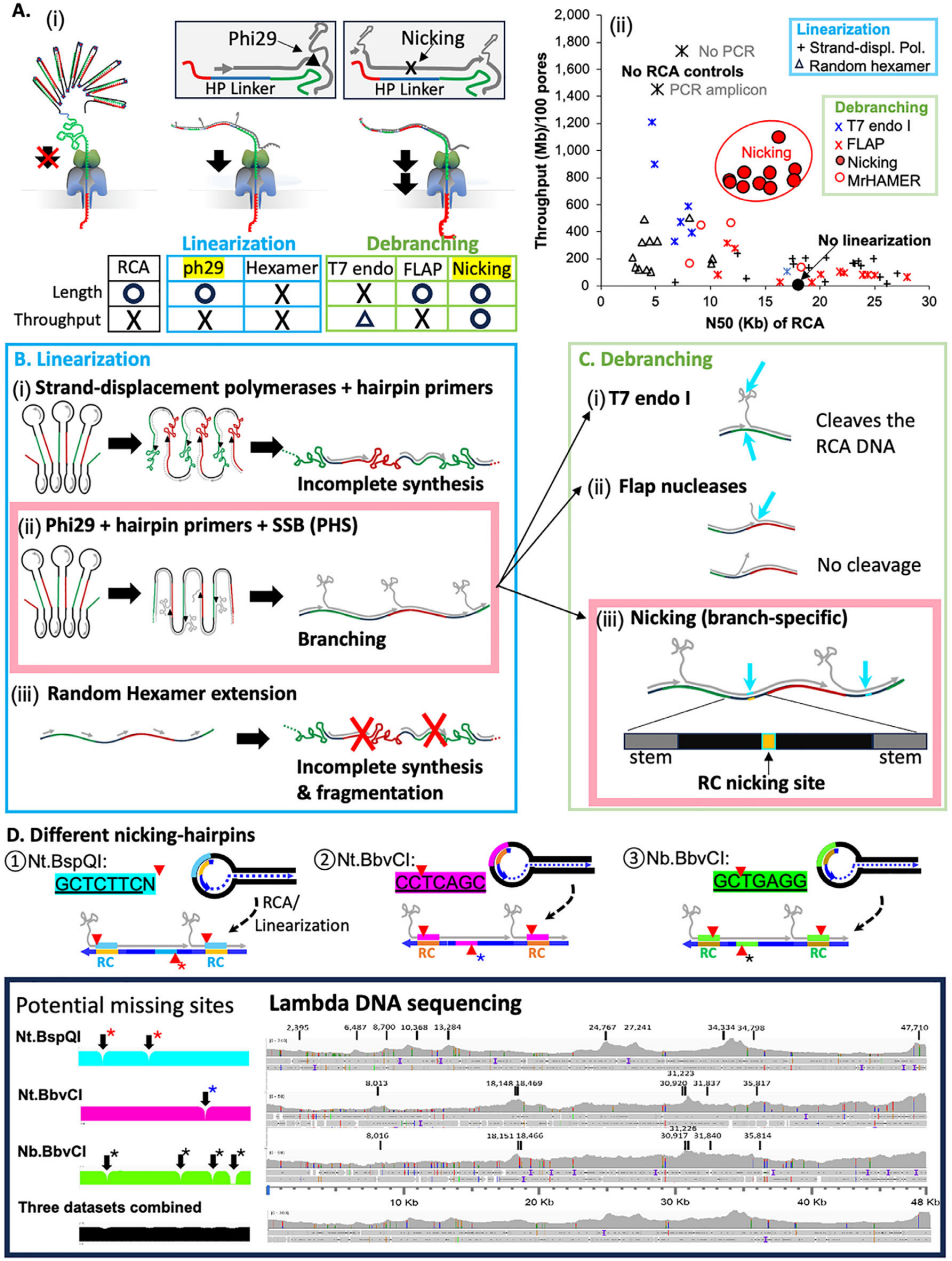
Linearization and debranching enhance RCA read length and throughput. A) Schematic view (‐*i*‐). Summary of read length (x‐axis) and throughput (y‐axis) after various linearization and debranching methods (‐*ii*‐). B) Linearization of RCA DNA by second‐strand synthesis was performed using hairpin primers (‐*i*‐ & ‐*ii*‐) or random hexamers (‐*iii*‐). The PHS (Phi29+hairpin primers+SSB) method was selected for the next step, debranching. Red crosses indicate template breakage. C) Three debranching methods were evaluated. Branch‐specific nicking (‐*iii*‐) specifically and effectively cleaves branched strands at hairpin locations. D) Hairpins with Nt.BspQI, Nt.BbvCI or Nb.BbvCI yields reverse‐complementary nicking sites in newly synthesized DNA (red arrow heads). The boxed panel shows the read coverage on lambda phage genomic DNA when using three different nicking hairpins. Arrows indicate the positions of nicking sites in lambda DNA. Combining all three datasets (bottom black) minimizes the probability of losing sequence coverage near nicking sites.

Surprisingly, RCA products showed an enrichment of subreads <1 Kb (Figure 1B,D‐*i*‐), despite negligible <1 Kb fragments in the input DNA (Figure 1A‐*i‐*). These subreads exhibited lower G/C content (Figure 1H) and abnormal concatemerization patterns, suggesting RCA recombination (Figure 2B‐*ii*‐).^[^ ^49^, ^50^ ^]^ RCA recombination is often caused by Phi29 stalling at inhibitory secondary structures (e.g., hairpins, G‐quadruplexes) on either the template^[^ ^51^ ^]^ or non‐template strands.^[^ ^52^ ^]^

### 2.2 Single‐Strand DNA‐Binding Proteins (SSBs) Improve RCA Performance

Although Phi29 is highly processive with double‐stranded DNA templates, it is less efficient on single‐stranded templates due to secondary structures, which induce polymerase stalling that often causes recombination.^[^ ^49^, ^50^, ^51^, ^52^ ^]^ We hypothesized that the read‐length bias results from Phi29 stalling during initial extension, when the template becomes partially single‐stranded and forms inhibitory structures (Figure 1B‐*ii‐*). Longer target DNA fragments are more prone to this effect.

SSBs, which reduce secondary structures of DNA templates, have been shown to improve DNA polymerase processivity, including Phi29,^[^ ^53^, ^54^ ^]^ and reduce RCA recombination.^[^ ^49^, ^54^ ^]^ Including SSBs in the reaction significantly reduced the proportion of <1 Kb subreads from 47.5 ± 6.8% to 26.5 ± 13.7% (*p* = 0.0434; Figure 1D‐*ii*‐,G).

### 2.3 Pre‐Circling Further Improves Read Length

Next, we tested whether converting hairpin‐ligated DNA into fully double‐stranded circular DNA (“pre‐circling”) prior to RCA would further reduce length bias (Figure 1C‐*ii*‐). A similar approach has been used for PacBio CCS. The pre‐circling step, using Sequenase and SSB, significantly reduced the <1 Kb subread fraction to 25.2 ± 6.9% (*p* = 0.0037) and increased subread N50 from 3825 ± 1282 to 4113 ± 714 bp (Figure 1C‐*ii‐*,D‐*iii*‐). It also improved RCA read lengths (Figure 1E; *p* = 0.0147) and modestly increased throughput as well (Figure 1F; Figure S1, Supporting Information).

### 2.4 Linearizing RCA DNA Is Critical for Nanopore Sequencing

Unlike PacBio, which detects indirect fluorescence signals of nucleotide extension on target DNA, Nanopore sequencing relies on the physical translocation of target DNA or RNA strands through a read pore. As such, its sequencing efficiency is likely sensitive to secondary structures of target molecules.^[^ ^55^, ^56^ ^]^ Our initial attempts to sequence long RCA DNA using standard Nanopore protocols nearly completely failed, yielding fewer than 1000 reads (“No linearization” control in **Figure** **2**A; Figure S2A, Supporting Information).

To improve read throughput, we tested multiple linearization strategies (Figure 2B), aiming to minimize fragmentation since longer RCA reads increase both consensus length and quality. We explored various DNA polymerases to synthesize complementary strands and reduce secondary structures, using either hairpin‐specific primers or random hexamers (Figure 2B; Figure S2B,C, Supporting Information). Most conditions resulted in poor read‐throughput and/or fragmented templates (Table S2, Supporting Information). However, Phi29 extension with hairpin primers in the presence of SSB (hereafter, PHS) achieved substantially improved read throughput (177.11 ± 23.75 Mb per 100 pores) and N50 of 23.17 ± 1.43 Kb (Figure 2A‐*ii*‐; Figure S2B, Supporting Information). Still, PHS throughput was ninefold lower than routine PCR amplicon sequencing (1450.91 ± 718.46 Mb per 100 pores; Figure 2A‐*ii*‐; Table S2, Supporting Information), limiting its utility for routine laboratory use.

### 2.5 Efficient Debranching Is Necessary to Further Improve Read Throughput

Given Phi29's high processivity, we hypothesized that branched DNA structures formed during PHS might interfere with Nanopore sequencing (Figure 2B‐*ii*‐). Linearizing structured DNA or RNA strands has been shown to dramatically improve read‐throughput.^[^ ^55^, ^57^ ^]^ We tested published debranching methods (Figure 2C; Figure S2D and Tables S1 and S2, Supporting Information). T7 endonuclease I treatment, used in R2C2,^[^ ^37^ ^]^ INC‐seq,^[^ ^38^ ^]^ and NanoAmpli‐Seq,^[^ ^39^ ^]^ significantly improved read throughput (646.49 ± 342.18 Mb per 100 pores; Figure 2A; Table S2, Supporting Information), but severely shortened RCA reads (N50 = 6.66 ± 1.54 Kb), suggesting non‐specific cleavage (Figure 2C‐*i*‐).

Next, we evaluated enzymes with Flap endonuclease activity (e.g., FEN1 and Taq polymerase)^[^ ^58^, ^59^, ^60^ ^]^ to more specifically cleave branched DNA. These yielded longer reads (N50 = 18.7 ± 5.4 kb) but did not improve throughput (158.39 ± 108.55 Mb per 100 pores; Figure 2A*‐ii*‐; Table S2, Supporting Information). The “Flap” activity is known to be inefficient for targets with unstable secondary structure (Figure 2C‐*ii*‐).^[^ ^60^ ^]^

To achieve both specific and efficient debranching, we employed target‐specific nicking endonucleases (Figure 2C‐*iii‐*). We incorporated a 7‐base recognition site for nickases—Nt.BspQI, Nt.BbvCI, or Nb, BbvCI—into our hairpin DNA (Figure 2D). Nickase treatment significantly improved throughput (818.42 ± 108.87 Mb per 100 pores) while maintaining long reads (N50 = 14.65 ± 2.21 Kb; Figure 2A‐*ii‐*; Table S2, Supporting Information). This represents a 2.8‐fold improvement over the methods used in MrHAMER^[^ ^40^ ^]^ and a 1.6‐fold improvement over T7 endonuclease I treatment.^[^ ^37^, ^38^, ^39^ ^]^

The likelihood of a random 7‐base recognition site appearing in DNA is roughly one in 16384 nts. By using all three nickase types, the probability of losing sequence coverage near nicking sites is further minimized (e.g., the chance of all three nicking sites occurring within a 100 nt region is ≈1.9 × 10^−7^), supporting the utility of our approach for sequencing long DNA and complex DNA pools (Figure 2D).

### 2.6 CLAE Improves Read Accuracy with or Without Reference

**Figure 3:**
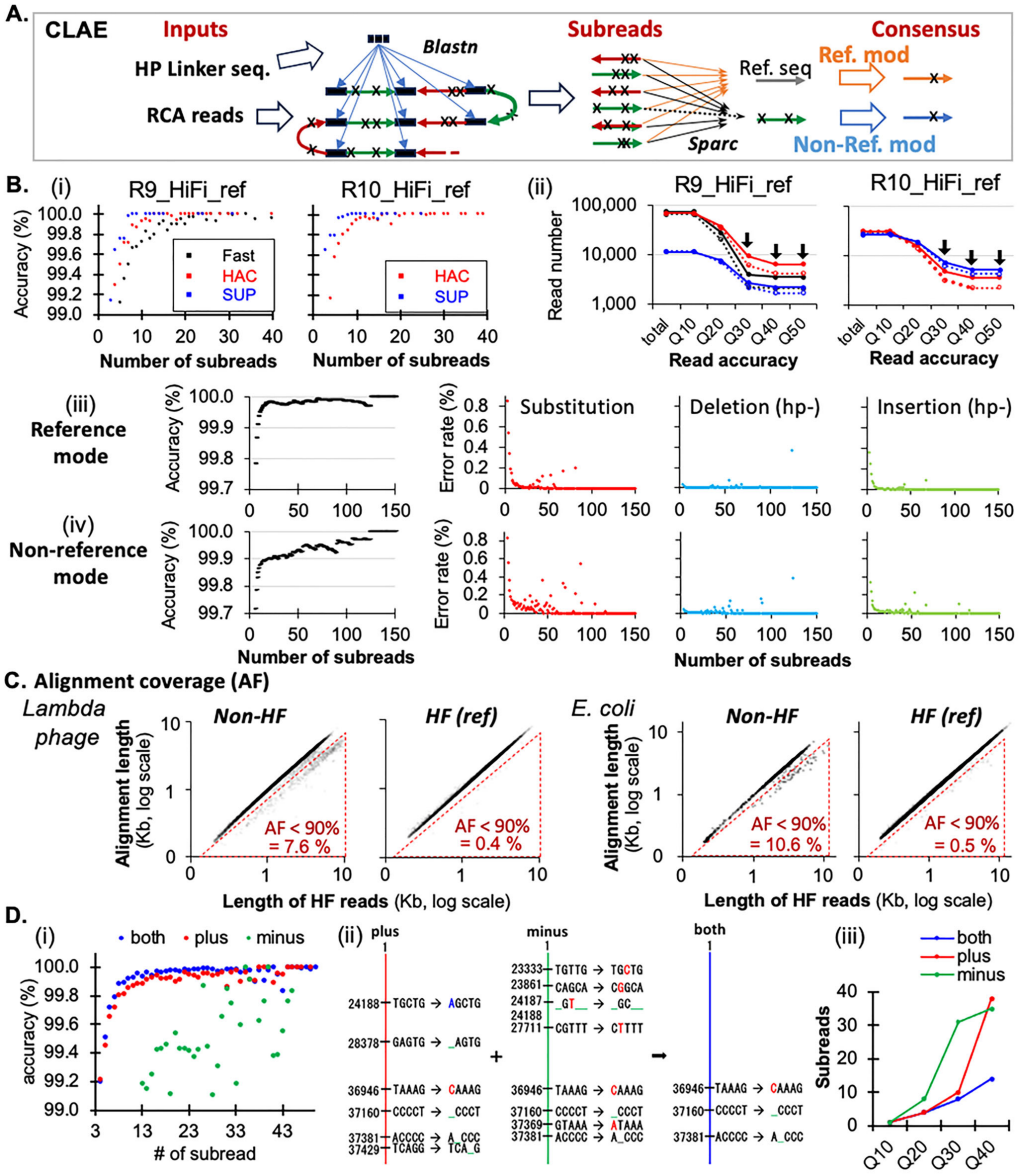
CLAE enables efficient error correction with or without a reference. A) Overview of CLAE's error correction workflow. B) Comparing base‐calling modes for R9 and R10 flow cells. Consensus accuracy (y‐axis) versus subread count (x‐axis) across base‐calling modes (‐*i*‐). Number of ≥Q30 HF reads (arrows) depends on base‐caller and flow cell (‐*ii*‐). Both reference‐based and non‐reference modes effectively reduce substitution, deletion, and insertion errors (‐*iii*‐). C) Alignment fractions of the shorter read (AF) for HF and raw (non‐HF) reads, shown for Lambda phage DNA (left) and *E. coli* genomic DNA (right). HF were generated with the reference mode (ref). Dotted triangles denote reads with <90% AF. D) Error correction efficiencies using “plus (red)” strands only, “minus (green)” strands only, and both “plus and minus” (blue) strands (‐*i*‐). Many errors occur at error‐prone k‐mers (‐*ii*‐). Using both strands reduces the subread threshold to achieve ≥ Q30 HF reads (‐*iii*‐).

Our CLAE pipeline (available at https://github.com/xdtdaniel/CLAE) extracts subreads from raw RCA reads and uses them to generate consensus sequences (**Figure** **3**A). Both reference‐guided and non‐reference modes significantly improved read accuracy relative to raw reads, exponentially reducing substitution, deletion, and insertion errors with increasing subread numbers (Figure 3B). Although homopolymer errors persisted (Figure S3, Supporting Information), these are computationally correctable.^[^ ^61^, ^62^ ^]^ Reflecting the improved read accuracy, almost all (99.5%) high‐fidelity (HF) reads exhibited nearly complete alignment coverage to reference (defined by >90% alignment fraction of the shorter read or “AF”), while a substantial portion of raw reads (7.6–10.6%) failed to show >90% AF (Figure 3C).

Moreover, using both forward (“+”) and reverse (“−”) strand subreads was more effective than using single‐direction subreads only for consensus generation (Figure 3D). Unlike our approach, methods based on self‐ligation‐mediated RCA produce only single‐direction subreads and suffer from limited consensus read accuracy.^[^ ^37^, ^38^, ^39^, ^40^ ^]^ This issue likely stems from error‐prone “k‐mers”,^[^ ^45^, ^46^ ^]^ which remained difficult to correct when using single‐direction subreads alone. Using both “+” and “‐” strands effectively corrected most of these errors (Figure 3D‐*ii*‐). Q40‐level reads were achieved when using 14 or more subreads of both “+” and “‐” strands, whereas it required >35 reads when using only single‐direction strands (Figure 3D‐*iii*‐).

### 2.7 Flow Cell Type and Base‐Calling Mode Affect Consensus Quality

We evaluated R9.4.1 and R10.4.1 flow cells using three base‐calling modes: SUP (super accuracy), HAC (high accuracy), and FAST (fast) (Figure 3B; Table S3, Supporting Information). R10.4.1 flow cells with double read‐heads exhibit higher read accuracy over R9.4.1 flow cells.^[^ ^44^ ^]^ At equivalent subread counts, SUP‐mode reads consistently produced the most accurate reads for both flow cell types (Figure 3B‐*i*‐), achieving ≥Q30 level accuracy for 24% ∼ 27% of pass reads—1.7‐ and 4.5‐fold higher than HAC or FAST, respectively (Table S3, Supporting Information).

However, the total number of high‐quality (Q30) HF reads depended on both the number and quality of pass reads. On R9.4.1 flow cells, HAC mode yielded 4.1‐fold more pass reads than SUP mode, resulting in 3.5‐fold more Q30 HF reads (see Figure 3B‐*ii‐* and its source data). For R10.4.1 flow cells, however, SUP mode exhibited improved pass read rates (only 1.5‐fold lower than HAC) and thereby produced 1.5‐ to 2.0‐fold higher numbers of ≥Q30 reads than HAC mode.

Overall, our data indicate mode‐specific differences between flow‐cell types generating ≥Q30 level HF reads. The optimal configuration was R9.4.1 with HAC mode (1574 HF reads per 100 000 RCA reads). R10.4.1 with SUP mode showed comparable efficiency (1188 Q30‐level HF reads per 100 000 RCA reads) (Figure 3B‐*ii‐* and its source data).

### 2.8 High‐Fidelity Long Reads Enable Sensitive Detection of Local SARS‐CoV‐2 Variants

The SARS‐CoV‐2 pandemic highlighted the need for rapid and accurate detection of local variants in community settings such as wastewater.^[^ ^11^, ^12^, ^13^, ^14^ ^]^ Identifying genetic variants in a mixed pool (e.g., wastewater) requires detecting the co‐occurrence of multiple variant‐specific signature mutations within the same RNA molecule. However, this has been challenging due to the limitations of frequent RNA fragmentations, short‐read sequencing, and inefficient PCR amplification.^[^ ^14^, ^63^, ^64^ ^]^ Detecting signature mutations spanning longer regions, beyond the reach of typical PCR amplicons (∼400 nt using ARTIC primers^[^ ^65^ ^]^), remains impractical using short‐read sequencing (100–200 nt reads).

**Figure 4:**
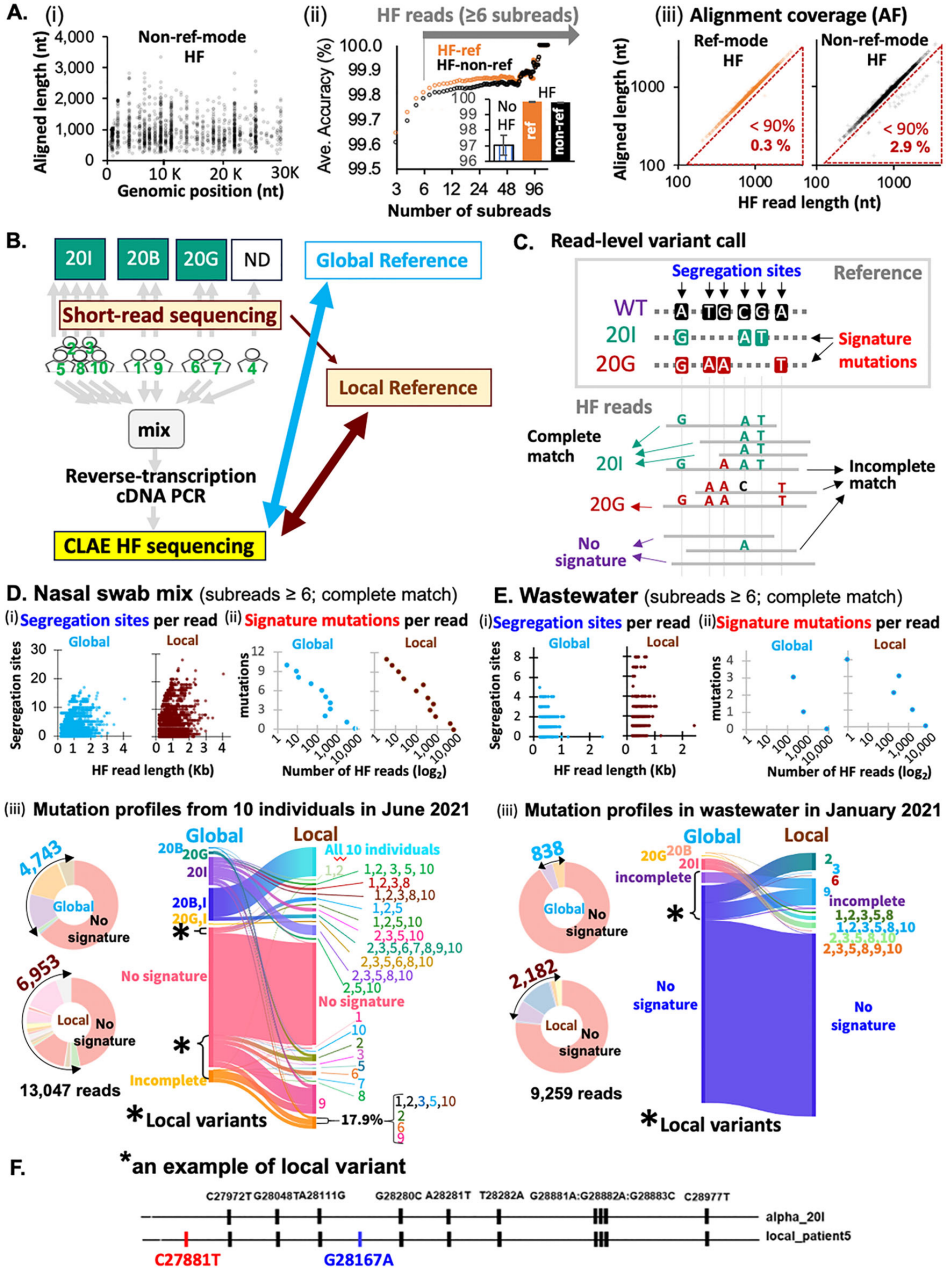
CLAE detects SARS‐CoV‐2 variants at the read level. A) The lengths of HF reads (y‐axis) plotted for 29 major target sites of the SARS‐CoV‐2 genome (x‐axis) (‐*i*‐). Read accuracy (‐*ii*‐) and alignment coverage (AF) (‐*iii*‐) of HF reads were compared for reference‐based (orange) or non‐reference modes (black). B) Study design for mixed‐sample sequencing and comparison to local/global references. Ten nasal swab samples, individually analyzed with short‐read sequencing, were mixed and subjected to HF Nanopore sequencing. HF reads were compared with both global references and local references. C) Variant assignment based on matching all segregation sites within each HF read. D,E) Variant profiles in nasal swab mix (D) and wastewater (E) compared against global (cyan) and local (brown) references. Longer HF reads have more segregation sites per read (D‐i, E‐i). Frequency of signature mutations (D‐ii, E‐ii). Asterisks mark HF reads matching local but not global references, indicating cryptic mutations (‐*iii*‐). F) Example of a read containing local mutations.

To address this, we used CLAE to evaluate the utility of high‐fidelity Nanopore sequencing for read‐level detection of viral variants in a mixed pool of SARS‐CoV‐2‐positive nasal swab samples (from 10 asymptomatic individuals, May–June 2021^[^ ^66^ ^]^ (Tables S4 and S5, Supporting Information). Short‐read sequencing of these samples (each sample individually) revealed known alpha variant mutation profiles (**Figure** **4**B). We also analyzed a wastewater sample from January 10, 2021 (SY_0 110 2021), collected from the Southerly Wastewater Treatment Plant in Columbus, Ohio (estimated 130 viral copies µL^−1^) as described previously.^[^ ^67^ ^]^

To maximize read lengths and sensitivity, we developed a multiplex reverse‐transcription method (Figure S4A, Supporting Information). Unlike ARTIC protocols that produce fixed‐length cDNA amplicons,^[^ ^65^ ^]^ our method generates variable‐length cDNAs using 132 reverse primers targeting 29 mutation‐rich SARS‐CoV‐2 regions (3–6 primers per region; Table S6, Supporting Information). For sensitive detection of cDNAs of varying lengths from these primers, the 3′ ends of cDNAs were ligated to hairpin linkers (HPA_6N3C6) containing a random hexamer splint. Consistent with previous reports using similar linkers,^[^ ^68^ ^]^ our linkers showed near complete ligation efficiency (≈90% ±12 SD) (Figure S4A, Supporting Information). The ligated cDNAs of varying lengths were then amplified using a PCR strategy optimized for long amplicons and minimal recombination (Figure S4A, Supporting Information).

The resulting DNA was subjected to CLAE and sequenced on R9.4.1 flow cells using HAC base‐calling mode. In control experiments with wild‐type SARS‐CoV‐2 RNA (NR‐52388 from BEI Resources), all 29 target regions were detected (Figure 4A‐*i*), with 99.777 ± 0.004% and 99.808 ± 0.008% read accuracy using non‐reference and reference‐based modes, respectively (Figure 4A‐*ii*). Most HF reads (97–99%) exhibited near full‐length coverage (>90% AF) to reference (Figure 4A‐*iii*).

For variant calling in nasal swab and wastewater samples, we used CLAE's non‐reference mode (≥6 subreads) to avoid potential bias introduced by reference‐based correction, which can reduce intrinsic sequence diversity.^[^ ^69^, ^70^ ^]^ We identified co‐occurring species‐specific signature mutations within each HF read to classify SARS‐CoV‐2 subspecies (Figure 4C). We used 70 segregation sites from COVID‐19 CG database^[^ ^71^ ^]^ to compare with the global alpha clade references (Table S7, Supporting Information) and 128 locally defined sites from our nasal‐swab Illumina sequencing data (Table S8, Supporting Information). A read was assigned to a specific variant only if it exactly matched all segregation sites it covered.

As expected, the number of segregation sites per read positively correlated with read length (Figure 4D‐*i*‐). The number of signature mutations per read ranged from 0 to 12 (with global reference) and from 0 to 14 (with local reference) (Figure 4D‐*ii‐*). Of all HF reads, 4743 (36.4%) completely matched global alpha clade references (Figure 4D‐*iii‐*), while 6953 (53.3%) matched local references from our nasal‐swab samples. The discrepancy suggests the presence of cryptic, locally circulating mutations not captured in global databases (Figure 4F).^[^ ^11^, ^12^, ^13^, ^14^ ^]^

### 2.9 Cryptic Mutations in Wastewater Suggest the Early Circulation of Local Variants

HF reads from wastewater samples were shorter than those from nasal swabs (Figure 4E‐*i*‐), consistent with increased RNA fragmentation in wastewater. In line with our nasal‐swab analysis, 23.6% of HF reads (2182 reads) matched local reference profiles, compared to only 9.1% matching global references (Figure 4E‐*iii‐*). These findings suggest that similar local variants were circulating in the community as early as January 2021. Notably, some mutations unique to our local alpha variants were identified in Delta and Omicron reference sequences that emerged in mid‐2021^[^ ^72^ ^]^ (Figure S4C, Supporting Information). These observations align with previous wastewater surveillance studies that detected potential precursor variants.^[^ ^12^, ^14^ ^]^ Cross‐contamination between wastewater and nasal swab samples is unlikely, as sequencing was performed in separate facilities and at different time points.

### 2.10 Agnostic Sequencing Profiles Novel RNA Viruses in the Ocean

Metagenomic studies have greatly expanded our understanding of the RNA virosphere, doubling the number of known viral phyla from five to at least ten.^[^ ^4^, ^5^, ^6^ ^]^ However, most of these discoveries rely on short‐read sequencing of a single gene marker, which limits genome completeness and hinders sequence‐based virus taxonomy.^[^ ^10^, ^73^ ^]^ To address these challenges, we evaluated the utility of CLAE for agnostic, long‐read sequencing of unknown RNA viruses in ocean samples.

**Figure 5:**
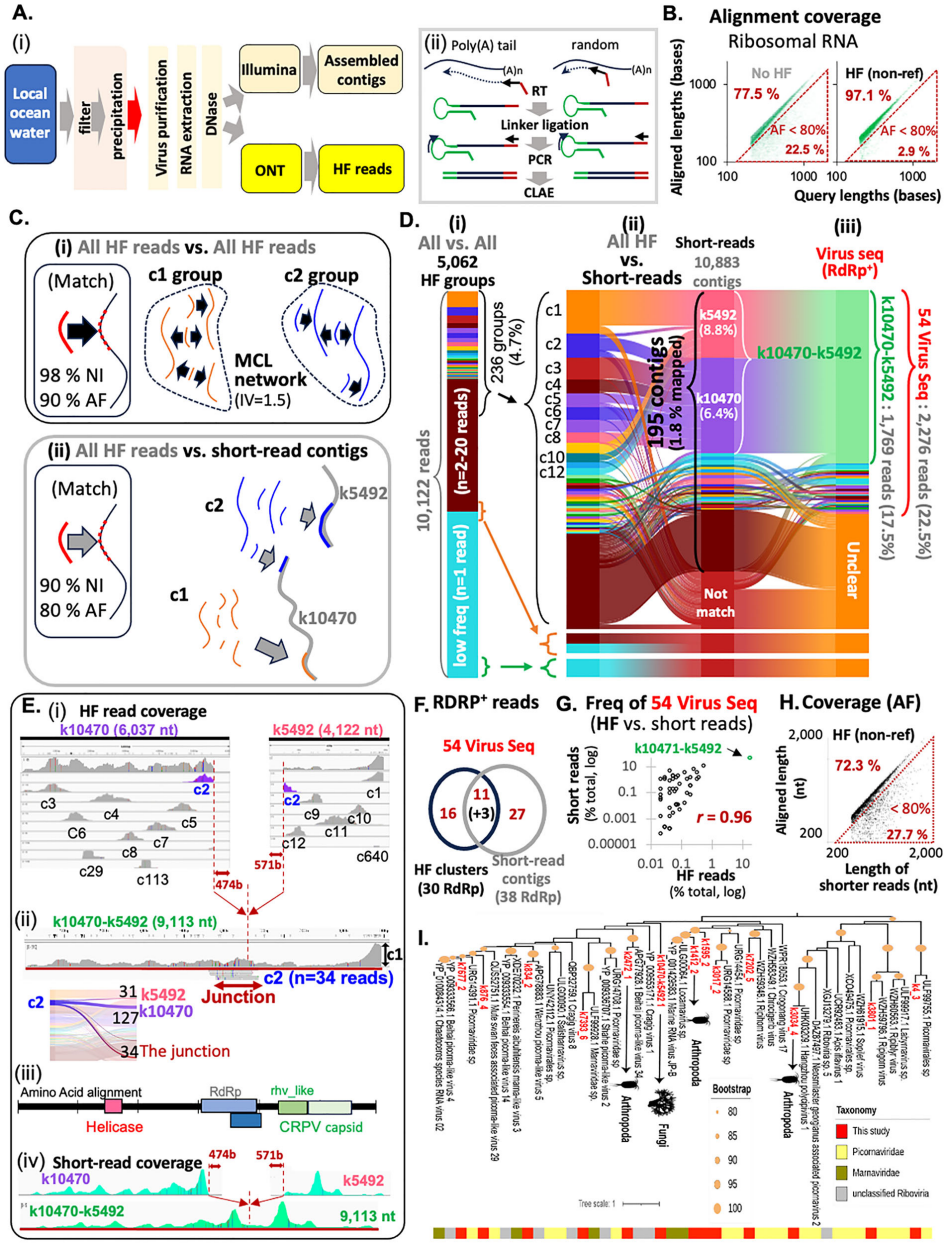
Agnostic HF sequencing identifies novel RNA viruses in ocean RNA. A) Workflow for viral particle enrichment and sequencing (‐*i‐*). cDNAs synthesized using poly(A)‐ or random primers (‐*ii‐*). B) Comparison of ocean HF rRNA reads to prokaryotic rRNA references. Red triangle: reads with AF <80%. C) HF reads were clustered by ≥98% identity and ≥90% AF into 5062 groups (‐*i*‐). Reads mapped to 10883 MEGAHIT contigs at ≥90% identity and ≥80% AF (‐*ii‐*) D). Size distribution of HF clusters (‐*i*‐); overlap of 236 HF groups with 195 short‐read contigs (‐*ii‐*); 54 RdRp‐containing virus sequences identified (‐*iii‐*). E) Mapping patterns of HF clusters to contigs k10470 and k5492 (‐*i‐*). c2 reads span both contigs, suggesting mis‐assembly (‐*ii‐*). A fused contig (k10470–k5492) was constructed (ii) and linked to “Beihai picorna‐like virus 55″ (iii). Short‐read coverage shown (iv). F) Virus sequences identified from both HF and short‐read datasets. Eleven short‐read contigs and 14 HF groups were common. G) Comparison of virus detection by HF reads (x‐axis) and short reads (y‐axis) (*r* = 0.96). H) HF reads and short‐reads were considered matching when AF is ≥ 80%. I) phylogenetic tree showing 13 virus sequences. Four RdRps could be assigned to putative hosts (arthropods and fungi).

Briefly, seawater was processed by size‐filtration and chemical flocculation of the filtrate, followed by washing the flocculated virion concentrates as previously described.^[^ ^3^, ^6^, ^74^, ^75^ ^]^ Extracted RNAs were sequenced using traditional short‐read sequencing as previously described^[^ ^3^, ^6^ ^]^ and Nanopore HF sequencing with CLAE protocols (**Figure** **5**A‐*i*‐). For Nanopore HF sequencing, ocean viral RNAs were reverse‐transcribed, and cDNAs were amplified and sequenced using similar methods described above, except that reverse‐transcription was performed using both poly(A)‐tail‐specific primers and random primers in separate reactions (Figure 5A‐*ii*‐).

This approach yielded 17104 HF reads (with at least three subreads). 10883 short‐read‐assembled contigs were generated using MEGAHIT.^[^ ^3^, ^6^ ^]^ Despite efforts to enrich for viral sequences during ocean sample processes, 40% of HF reads were ribosomal RNA (rRNA, Figure 5B; Figure S5A, Supporting Information), leaving 10122 HF reads as putative RNA virus candidates. These were clustered into 5062 groups using a ≥98% nucleotide identity (NI) and >90% AF threshold (Figure 5C,D‐*i*‐; Figure S5B, Supporting Information). Group sizes ranged widely: the most abundant cluster (c1) had 298 reads (2.94% of total), while 4348 clusters (86%) contained a single read (Figure 5D‐*i*‐).

To assess CLAE's utility for detecting and quantifying novel RNA viruses, we compared HF reads to 10883 short‐read‐derived contigs generated with established methods.^[^ ^3^, ^6^ ^]^ Using species‐level operational taxonomic unit (OTU) criteria (≥90% NI and ≥80% AF) previously optimized for ocean samples^[^ ^6^ ^]^ (Figure 5C‐*ii‐*), 236 HF groups (4.7%) aligned to 195 short‐read contigs (1.8%) (Figure 5D‐*ii*‐). The limited overlap likely reflects the shallow sampling depth of highly diverse RNA viromes, rather than methodological bias. Low overlap among different ocean RNA virus datasets has also been reported previously.^[^ ^4^, ^5^, ^6^ ^]^

Interestingly, the top 12 most abundant HF groups (c1–c12) all aligned to just two short‐read contigs: k10470 (6.0 Kb) and k5492 (4.1 Kb). Of these, the alignment patterns of cluster c2 suggested potential mis‐assembly of these two contigs (Figure 5E). Specifically, one subgroup of c2 reads aligned 571 nt downstream of k5492's 5′ end, another aligned 474 nt upstream of k10470's 3′ end, and a third set spanned a putative junction between the two. (Figure 5E; Figure S5B, Supporting Information). These observations suggest that k10470 and k5492 originate from the same RNA virus genome. Short‐read coverage at the potential mis‐assembly sites was notably sparse compared to adjacent regions, supporting the mis‐assembly hypothesis (Figure 5E‐*iv*‐; Figure S5C, Supporting Information). Based on these findings, we generated a fused contig (k10470‐k5492), representing a likely full viral genome (Figures 5E‐*ii*‐). Approximately 17.5% of total HF reads were accounted for by the fused k10470‐k5492 (Figure 5D‐*iii*‐).

BLAST analysis revealed that the fused k10470‐k5492 sequence shares 75% genome coverage with “Beihai picorna‐like virus 55” (e‐value: 8e−126) (Figure S5D, Supporting Information). This sequence exhibits a dicistronic architecture, encoding two open reading frames (ORFs): one encoding a helicase and RdRp and the other for a capsid (Figure S5D, Supporting Information). Amino acid sequences encoded by these genes showed 35.9%, 40.0% and 34.3% identity to picornaviridae (95% coverage with e‐value of 4e‐25), marnaviridae (95% coverage with e‐value of 1e‐78), and picornaviridae sequences (87% coverage with e‐value of 2e‐119), respectively (Figure S5E, Supporting Information).

To identify additional viral sequences, we used an iterative, HMM search‐and‐update pipeline designed to capture even the most divergent RNA‐directed RNA polymerase (RdRp) sequences.^[^ ^6^ ^]^ We identified a total of 543 HF reads (5.4% of total) representing 30 distinct HF groups containing≥100 RdRp amino acid sequences, the detection threshold determined in prior studies.^[^ ^6^ ^]^ A total of 706 short‐read contigs (6.5%) also passed this threshold. The higher RdRp sequence rates in short‐read contigs reflect the read length differences (short‐read contigs: 502.8 nts ± 296.7 SD vs HF: 358.2 nts ± 163.2 SD), as detecting >100 RdRp amino acids requires ≥300 nt. Of these, 38 short‐read contigs matched to 41 HF groups with ≥90% NI and ≥80% AF.

In total, 54 unique virus sequences were identified—30 from HF groups and 38 from short‐read contigs, with 11 common to both datasets (3 HF groups aligned redundantly to the same contigs) (Figure 5F). Together, these virus sequences accounted for 22.5% of total HF reads. Detection frequencies for individual virus sequences were highly correlated between Nanopore and short‐read sequencing (*r* = 0.96; Figure 5G).

Phylogenetic inference based on our sequences and reference NCBI sequences clustered 11 sequences with *Picornaviridae* and *Marnaviridae* families, which belong to phylum *Pisuviricota* (i.e., Branch 2) in the global RdRp phylogenetic tree^[^ ^6^ ^]^ (Figure 5I). Eight (k4_3, k3801_1, k3034_4, k7202_5, k3017_2, k876_4, k7677_2, k10470‐k5492_1) and three (k1595_2, k1412_2, k7393_6) RdRps clustered with reference sequences of the families *Picornaviridae* and *Marnaviridae*, respectively. Two (k834_2, k2472_1) clustered with unclassified reference sequences identified as unclassified *Riboviria*, although likely members of the order *Picornavirales*. Abundance of these species is consistent with previous reports of marine RNA viromes^[^ ^6^, ^76^ ^]^ enriched in “pircorna‐like” single‐strand RNA viruses, suggesting that the novel strains identified here may infect marine unicellular eukaryotes (protists, fungi) and zooplanktonic invertebrates (Figure 5I).

## 3 Discussion

We developed CLAE, a high‐fidelity Nanopore sequencing strategy that overcomes key limitations of current high‐fidelity methods. While prior ONT fidelity‐enhancement methods have largely focused on consensus generation, CLAE resolves two key upstream challenges: i) the persistent read‐length bias caused by inefficient circularization and RCA, and ii) limited sequencing throughput due to suboptimal debranching or excessive DNA fragmentation (Table S1, Supporting Information). By systematically addressing these limitations through biochemical optimization and nickase‐based processing, CLAE achieves higher fidelity, longer reads, and improved applicability, suitable for both targeted and agnostic RNA virus sequencing and metaviromic analysis of environmental RNA samples.

Beyond error correction, RCA is increasingly used in biosensing, molecular diagnostics, and other sequencing‐based applications.^[^ ^41^, ^42^, ^43^, ^77^ ^]^ The optimizations described here also address long‐standing concerns in these areas, particularly RCA bias against structured or long templates—suggesting broader utility beyond Nanopore sequencing.

### 3.1 CLAE Enables Sensitive Profiling of RNA Viruses in Environmental Samples

In the context of SARS‐CoV‐2 surveillance, CLAE enabled the direct observation of co‐occurring mutations within individual viral RNA molecules, allowing accurate identification of quasispecies at the read level. Notably, CLAE detected cryptic mutations in wastewater samples that matched variants observed in nasal swabs collected five months later, highlighting its potential for early detection of locally circulating strains. These findings support CLAE as a valuable tool for real‐time viral surveillance and variant tracking.

CLAE also demonstrated strong performance in agnostic viral discovery. By applying the method to ocean RNA samples, we identified 54 putative RNA virus sequences using RdRp‐based homology searches. The most dominant virus, k10470‐k5492, accounted for 17.5% of all HF reads and was assembled by correcting a misassembly involving two short‐read contigs. Although it shares similarity with “Beihai picorna‐like virus 55,” its host remains unknown. Given the ecological significance of RNA viruses in marine ecosystems, further investigation into their host range and biology is warranted. Importantly, viral sequence frequencies derived from CLAE correlated well with those from short‐read sequencing, suggesting that CLAE provides not only qualitative but also quantitative insights for metagenomic viral ecology.

### 3.2 Limitations and Opportunities for Improvement

While CLAE improves the accuracy of ONT sequencing, some limitations persist. Homopolymer‐associated indel errors remain a challenge, though they are computationally correctable. Compared to PacBio CCS, CLAE's consensus read lengths and throughput remain lower, especially for long RCA DNA templates requiring three or more subreads. Future work optimizing enzymatic processivity and automation could further improve performance.

The inherent fragility of RNA and its tendency to fragment in environmental samples remains a major hurdle.^[^ ^8^, ^14^, ^63^, ^64^ ^]^ Given Nanopore's relatively lower throughput compared to short‐read or PacBio platforms, improving cDNA yield and length is essential for successful environmental virome profiling. Our strategy—employing a novel hairpin linker (HPA_6N3C6)—achieves high ligation efficiency (>90%) and allows reverse transcription of cDNAs of any length. The HPA_6N3C6 linker contains a random hexamer splint and, through this, efficiently ligates to the 3′end of single‐stranded cDNA of any length. These types of DNA linkers exhibit near‐complete ligation efficiency.^[^ ^68^ ^]^ By maximizing amplicon lengths through optimized PCR, we successfully recovered thousands of viral sequences from minimal input RNA. In contrast to protocols like ARTIC,^[^ ^65^ ^]^ which rely on fixed‐length (≈400 nt) amplicons and may miss variants or small viral RNAs lacking primer‐binding sites, CLAE preserves long‐range mutation linkage and captures a broader spectrum of viral diversity.

In summary, CLAE represents a significant advance in high‐fidelity Nanopore sequencing. It enables read‐level variant detection, improves genome completeness in de novo viral discovery, and broadens the applications of portable sequencing for genomic surveillance. By bridging the accuracy gap between ONT and traditional platforms while maintaining real‐time, cost‐effective, and field‐deployable advantages, CLAE offers a versatile framework for applications in metagenomics, environmental virology, epidemiology, and clinical diagnostics.

## 4 Experimental Section

### *E. coli* gDNA and Lambda Phage DNA Preparation

Genomic DNA from E. coli strain K12 (NEB C2987PVIAL) was extracted using QIAGEN Genomic‐tip 500/G (10 262) & Genomic DNA Buffer Set (19 060) according to the manufacturer's protocol. Bacteriophage lambda DNA (cI857*ind* 1 *Sam* 7) was purchased from NEB (NEB N3011). The genomic DNA was fragmented using a g‐Tube (Fisher Scientific NC0380758) with the 20 Kb target condition, involving centrifugation at 4200 rpm for 2 mins using an Eppendorf 5424. The DNA was then concentrated with an Amicon Centrifugal Filter, 100 kDa MWCO (Sigma UFC510096), following the manufacturer's instructions. For an RCA reaction, DNA was end‐repaired and A‐tailed using NEBNext FFPE Repair (NEB M6630) and NEBNext Ultra II End Prep kit (NEB E7546S) according to the manufacturer's instructions.

### RCA‐Hairpin Ligation to A‐Tailed Double‐Stranded DNA

To generate hairpin linkers, hairpin oligo (“Hairpin1_NtBspQI”, “Hairpin1_NtBbvCI”, or “Hairpin1_NbBbvCI”, see Table S6, Supporting Information for sequence information) was incubated at 95 °C for 2 min followed by snap cooling in icy water (at the final concentration of 15 µm in 10 mm Tris‐EDTA Buffers). The hairpin linkers with 5′end phosphorylated “T” (final concentration 0.40 µm) were then ligated to the A‐tailed double‐stranded template DNA using the NEBNext Ultra II Ligation Master Mix (NEB E7595) according to the manufacturer's protocol. The ligation reaction was incubated at 20 °C for 1 h and then inactivated by incubating it at 65 °C for 20 min. Unligated, or partially ligated hairpin adapters were digested with 200 units of ExoIII (NEB M0206), 20 units of ExoVIII (NEB M0545), and 10 units of Lambda Exonuclease (NEB M0262) in 1X rCutSmart Buffer at 37 °C for 1 h, followed by heat inactivation at 80 °C for 20 min. Hairpin‐ligated DNAs were then purified using AMPure XP beads (Beckman Coulter A63880) at 0.45× bead volume and eluted in 40 µL of 10 mm TE buffer.

The efficiency of RCA‐Hairpin ligation to double‐stranded DNA was measured using PCR amplified DNA spanning 5945 to 6129 nt of NL4‐5 HIV‐1. The control DNA was amplified using NL5945F and NL6129R primers (see Table S6, Supporting Information for sequences) and Premix Taq DNA Polymerase Hot‐Start Version (Takara R028A). The RCA‐hairpin linkers were ligated to PCR‐amplified DNA as described above. The copy numbers of total DNA and the hairpin‐ligated DNA were measured by digital PCR (QuantStudio 3D Digital PCR) using ligated‐DNA‐specific primer sets (NL5961F and Sequencing_NtBspQI) and primers that detect all cDNAs (NL5961F and NL6034R). Oligonucleotide probes that align to the NL4‐3 5983 position (NL5983F_VIC) were used for digital PCR.

### Pre‐Circling of Hairpin‐Ligated DNA

Hairpin‐specific RCA primers (“Sequencing_NtBspQI”, “Sequencing_NtBbvCI”, or “Sequencing_NbBbvCI”, see Table S6, Supporting Information for sequence information) were conditioned by heating to 80 °C for 2 min and quickly cooled down to 4 °C using the SimpliAmp thermocycler (AppliedBiosystems). The RCA primers (final concentration of 5 µm) were mixed with up to 1 µg of hairpin‐ligated template DNA in a 50 µL reaction and incubated at 20 °C for 1 h. The extension of template‐bound primers was carried out with the following components and final concentration: 52 units of Sequenase Version 2.0 DNA polymerase (Applied Biosystems 70775Y200UN), 1 µg SSB (NEB M2401) in 1X Sequenase Reaction Buffer at 37 °C for 1 h. The reaction was heat‐inactivated at 65 °C for 10 min. Pre‐circularized DNAs were then purified using AMPure XP beads (Beckman Coulter Cat. # A63880) at 0.45× bead volume and eluted in 40 µL 10 mm TE buffer.

### Rolling Circle Amplification (RCA)

Pre‐circularized template DNA was then amplified by RCA using EquiPhi29 (ThermoFisher A39392). A 50 µL RCA reaction, which includes template DNA (3 ng to 1 µg), 1× EquiPhi29 Buffer, dNTPs (1 mm), DTT (1 mm), 0.5 U µL^−1^ EquiPhi29 Pol, and 20 ng µL^−1^ SSB) was incubated at 42 °C for 3 h. Following this, an additional 50 µL of the same RCA reaction mixture (except that the template DNA was substituted with nuclease‐free water) was added to the reaction and incubated for another 3 h at 42 °C. The reaction was stopped by incubating at 65 °C for 15 min and stored at 4 °C. RCA high‐molecular‐weight (HMW) DNA was size‐selected using the short‐read eliminator kit (Circulomics SS‐100‐101‐01), according to the manufacturer's protocol, and eluted in 30 µL of 10 mm TE buffer.

### Linearizing HMW RCA DNA Product

The primers specific to the hairpin for the second‐strand synthesis (“SecStrand_NtBspQI”, “SecStrand_NtBbvCI”, or “SecStrand_NbBbvCI”; see Table S6, Supporting Information for sequence information) were conditioned by heating to 80 °C for 2 min and quickly cooled to 4 °C using the SimpliAmp thermocycler (AppliedBiosystems). The primer (6 µm final concentration) was annealed to 2 µg of the purified HMW RCA DNA product by a 1 h incubation at 20 °C in the presence of 1x EquiPhi29 Reaction buffer. Second‐strand synthesis was carried out by extending the annealed primers at 42 °C for 1 h in a 50 µL reaction with the following components and final concentrations: 2 µg of primer‐bound HMW RCA DNA, 1× EquiPhi29 Buffer, dNTPs (1 mM), DTT (1 mm), EquiPhi29 Pol (0.5 U µL^−1^), and SSB (20 ng µL^−1^). The reaction was heat‐inactivated by a 15‐min incubation at 65 °C. The linearized DNA was purified using AMPure XP beads (Beckman Coulter A63880) at 0.45× bead volume and eluted in 40 µL of 10 mm TE buffer.

### Debranching HMW RCA DNA by Nicking the Second‐Strand DNA

Linearized RCA DNA (up to 1.5 µg) with Nt.BspQI‐, Nt.BbvCI‐, or Nb.BbvCI‐hairpin sequences were then digested with 10 units of Nt.BspQI (NEB R0644S), Nt.BbvCI (NEB R0632S), or Nb.BbvCI (NEB R0631S) nicking enzymes in a 50 µL reaction with enzyme‐specific buffers (1× NEB r3.1 buffer for Nt.BspQI or 1x rCutsmart for Nt.BbvCI and Nb.BbvCI). The reaction was subjected to a 1‐h incubation at 50 °C for NT.BspQI or 37 °C for Nt.BbvCI, or Nb.BbvCI, followed by AMPure XP bead (Beckman Coulter A63880) cleanups at 0.45× bead volume. The DNA was then eluted in 40 µL of 10 mm TE buffer.

### Nanopore Sequencing and Base‐Calling Using R9 or R10 Flow Cells

For R9 flow cell analysis, debranched DNA samples were subjected to the Nanopore DNA library prep using the Ligation Sequencing Kit (SQK‐LSK110). All samples were then sequenced with MinION R9.4.1 flow cells. Reads were basecalled with Guppy basecaller 6.5.7 using dna r9.4.1 450 bps hac.cfg configuration file. For R10 flow cell analysis, the Ligation Sequencing Kit (SQK‐LSK114) was used. All samples were sequenced with MinION R.10.4.1 flow cells. Reads were basecalled with Dorado basecaller 0.7.3 using dna r10.4.1 400 bps hac.cfg configuration file.

### Reverse Transcription of SARS‐CoV‐2 RNA

Nasal swab samples were collected by the Ohio State University Wexner Medical Center from 10 COVID‐positive individuals between May and June 2021 (Tables S4 and S5, Supporting Information). The RNA viruses were purified and processed for Illumina sequencing at the Infectious Diseases Institute of Ohio State University as previously described^[^ ^66^ ^]^ (Figure S4, Supporting Information), and the 10 nasal RNA samples were equally mixed and further processed for Nanopore sequencing. Wastewater RNA samples were collected at the Southerly wastewater treatment plant, Columbus (SY_0 110 2021, and purified and tested for SARS‐CoV‐2 RNA using qPCR as described previously.^[^ ^67^ ^]^ SARS‐CoV‐2 control RNA (NR‐52388; 2019nCoV/Hong Kong/VM20001061/2020) was obtained from the BEI resources (https://www.beiresources.org). Reverse transcription was carried out with SuperScript IV Reverse Transcriptase (Life Technologies 18 090 050) and a total of 132 virus‐specific RT primers. Briefly, in a 13 µL reaction, primers (0.1 µm) were annealed to 50–100 ng of template RNA in the presence of dNTPs (0.8 mm each dNTP) by incubating at 65 °C for 5 min, followed by snap cooling to 4 °C for 2 min. Then, the following reaction mixture (7 µL), containing RT enzyme, was added to the 13 µL of the RNA‐primer mixture: 2.9× Reaction Buffer, DTT (14 mM), Actinomycin D (17 ng µL^−1^), SUPERase In RNase Inhibitor (2.9 U µL^−1^) (Life Technologies AM2694), and SuperScript IV Reverse Transcriptase (28 U µL^−1^). The reaction was incubated at 50 °C for 0.5 h. After the initial incubation, 20U of TGIRT‐III Enzyme (InGex TGIRT50, 20 U µL^−1^) was added to the reaction and incubated at 60 °C for 1 h for an additional extension of cDNA. Reactions were stopped by adding 1 µL of 5 m NaOH (Final conc. 0.25 mm µL^−1^) and incubated at 95 °C for 3 min. RNA digestion with NaOH was neutralized by adding 1 µL of 5 m HCl to the reaction and purified with DNA Clean & Concentrator (ZymoResearch D4014), according to the manufacturer's protocols and eluted in 40 µL of 10 mm TE buffer.

### Hairpin Ligation to the 5′ End of cDNAs

A total of 10 µm of *HPA_6N3C6* hairpin oligo (IDT Nuclease‐Free Duplex Buffer 1 072 570; see Table S6, Supporting Information for the sequence) was self‐annealed by denaturing at 95 °C for 3 min and cooling down with ramp rate 0.1 °C sec^−1^ to 50 °C and incubating for 10 min. The self‐annealed *HPA_6N3C6* hairpin adaptors were then ligated to the reverse‐transcribed cDNA in a 235 µL reaction with the following components and final concentrations: 0.5x T4 DNA ligase buffer (NEB), 0.1 mg mL^−1^ BSA (NEB), 4.6 µL of Quick T4 DNA ligase (NEB), and 0.65X Quick ligase buffer (NEB). After a 5‐min incubation at room temperature, 160 µL of PEG‐DMSO mix containing 2.5x T4 DNA ligase buffer, 50% DMSO (Sigma‐Aldrich), and 20% PEG‐8000 (Sigma‐Aldrich) was added to the hairpin‐cDNA ligation mix. The final mix was incubated at 22 °C for 2 h and then at 30 °C for 1 h. Hairpin‐ligated cDNA was then purified by phenol‐chloroform extraction with a final elution of 20 µL in 10 mm TE buffer.

The efficiency of Hairpin ligation to the 5′ end of cDNA was measured by digital PCR using synthetic RNA (HIV426‐515 that spans NL4‐3 HIV‐1 positions 426–515; see Table S6, Supporting Information). The synthetic RNA was reverse‐transcribed using poly(A)‐targeting primers (R17‐10dT) and followed the same Hairpin ligation steps described above. The copy numbers of total cDNA and the hairpin‐ligated cDNA were measured by digital PCR (QuantStudio 3D Digital PCR) using ligated cDNA‐specific primer sets (HPT and NL509R) and primers that detect all cDNAs (NL454F and NL509R). Oligonucleotide probes that align to the NL4‐3 490 position (NL490R_VIC) were used.

### Amplification of SARS‐CoV‐2 cDNAs

Amplification of hairpin‐ligated DNA was carried out via three‐step PCRs (Figure S4A,B, Supporting Information) in 50 µL reactions with the following components and final concentrations: 5 U µL^−1^ LA Taq Polymerase (Takara RR042), 10 U µL^−1^ SD polymerase (Boca Scientific 108 910), 0.5 µm for each primer (or primer mix). SimpliAmp Thermal Cycler from AppliedBiosystems was set for the following conditions: 94 °C for 1 min, cycling of 98 °C for 10 sec, 58 °C for 30 sec, and 68 °C for 4 min, and final extension of 68 °C for 18 min. The hairpin‐ligated cDNAs were initially amplified for 10 cycles using HPTloop_17b and R17b primers (1st PCR). These amplified products were column‐purified and eluted in 20 µL of 10 mm TE buffer. The elute was then divided into three separate reactions for the 2nd PCR with HPTloop_21b and different SCoV2 2nd PCR R mixes (A, B, or C) and amplified for 18 cycles (The primer sets for these PCR sets can be found in Table S6, Supporting Information). Each of these 3 sets amplifies cDNAs of specific genomic locations, designed to avoid the potential interference of primers that align to the neighboring target sites. This strategy, thereby, enables a PCR amplification of long cDNA that spans beyond the neighboring primer binding sites, while reducing potential PCR recombination. The amplicons from the 3 sets of 2nd PCR were column‐purified separately and eluted in 20 µL of 10 mm TE buffer. The products from the A, B, or C mix were then amplified separately by a 3rd PCR with HPTloop_22b and the corresponding sets of SCoV2 3rd PCR R mix (A, B, or C) for 8 cycles. Following a final column purification, the A, B, and C amplicons were mixed equally based on Nanodrop or Qubit quantities. Amplified DNAs were subjected to the CLAE procedure for Nanopore sequencing using R9.4.1 flow cells, base‐called with the “HAC” mode.

### Ocean RNA Collection, Purification, and Short‐Read Sequencing

Samples enriched in viral particles from ≈ 5 L of North Carolina coastal seawater were obtained by sequential filtration and chemical flocculation based on John et al. 2011,^[^ ^78^ ^]^ followed by washing by centrifugal ultrafiltration with Amicon devices MWCO 100 kDa and purification of viruses through CsCl density gradients.^[^ ^79^ ^]^ RNA was extracted from viruses using the Quick RNA viral kit (Zymo R1034), and contaminating DNA was digested with two rounds of TURBO DNase (Invitrogen AM2238). The extracted RNAs were then subjected to both Illumina sequencing and CLAE‐mediated HF long‐read sequencing (Figure 5A‐*i*‐). Detailed procedures for the Illumina sequencing and metatranscriptome assembly using Megahit were described previously.^[^ ^6^ ^]^

### HF Nanopore Sequencing of Ocean RNA

Reverse transcription was carried out using either poly(A)‐targeting oligos (R17‐10dT) or random oligos (R17‐6N) (Figure 5A‐*ii*‐). Reaction conditions for reverse transcription and hairpin‐ligation to cDNA are the same as described above for SARS‐CoV‐2 RNA analysis. The hairpin‐ligated DNA was amplified via PCR using LA Taq Polymerase (5 U µL^−1^, Takara RR042) and SD polymerase (10 U µL^−1^, Boca Scientific 108 910), with 10 µm HPTloop_17b and 10 µm R17b primers. The thermal cycling conditions were as follows: 94 °C for 1 min, followed by 25 cycles of 98 °C for 10 sec, 58 °C for 30 sec, and 68 °C for 12 min, with a final extension at 68 °C for 18 min. The amplicons from PCR were column‐purified and eluted in 20 µL of 10 mm TE buffer. Amplified DNAs were subjected to the CLAE procedure for Nanopore sequencing using R9.4.1 flow cells, base‐called with the “HAC” mode.

### CLAE

The detailed step‐by‐step instructions and toy datasets for CLAE are available on GitHub (https://github.com/xdtdaniel/CLAE). Briefly, the CLAE toolkit is composed of (A) subread extraction using anchor sequences and (B) *c*onsensus sequence generation steps. (A) subread extraction using anchor sequences: The positions of concatenated (repeated) subreads in HMW RCA DNA reads were identified by BLAST mapping of anchor sequences (e.g., repeated hairpin or PCR primer sequences), and individual subreads were extracted using blast_generator.py. For example, the following anchor sequences were used (multiple anchor sequences can be used) to extract subreads from RCA reads for lambda phage DNA harboring Nt.BspQI hairpins: >Hairpin_Nt.BspQI(TAACATTGCAATGTTGGCTTGCTCTAAGCTCTTCACTCAGATATGCTGATACAGAGCAAGCCAACATTGCAATGTTAT); >hairpin (GTTCTACTAAACCGTGTCAATCAGTGT); >hairpin‐stem (CCAAGAAACATAAACAGAACG); and >PCR primer (CTGATGCATTGAAGCTGGT). *(B)* Consensus Sequence Generation steps: this step uses the “sparc” algorithm^[^ ^80^ ^]^ by default to generate consensus sequences and operates in both “reference‐mode” and “non‐reference‐mode” modes. As an option, the “PBDAG‐Con” algorithm^[^ ^81^ ^]^ is also provided. The “reference” mode generates consensus sequences by comparing the extracted subreads to known reference, and the “non‐reference” mode selects a “seed read” within a group of comparing subreads and uses it as a reference to generate a consensus sequence of the group. The seed reads are the longest subreads within the extracted subreads from a single RCA read. The Python file “clae.py” coordinates the execution of these two sub‐steps.

### SARS‐CoV‐2 Variant Call

We used HF reads generated with CLAE's non‐reference mode and selected for the consensus reads generated with at least 6 subreads. These HF reads were then mapped to SARS‐CoV‐2 reference read (NC045512.2) using minimap2 [Li, 2021 #16 612} with an option ‐a ‐L –cs = long –secondary = no. The mismatch nucleotides for each read were then compared to the nucleotide sequences at 70 segregation sites of globally spread lineages (Table S7, Supporting Information), or 128 segregation mutations of our own local reference data of nasal swab samples (Table S8, Supporting Information). Global VOC reference was downloaded from the “lineage reports” section of COVID‐19 CG (covidcg.org)^[^ ^71^ ^]^ with options of “Mutation Type: NT” and “Consensus Threshold: 0.7”. The lineage type was determined only when all segregation site nucleotides between the HF read and a lineage type in comparison were fully matched (complete match); otherwise, the reads were defined as “Incomplete match” or “No signature” (Figure 4C).

### Ocean RNA Analysis

We generated Nanopore HF reads with ≥ three subreads using CLAE as described above. rRNA reads were identified using the default options of SortMeRNA (v4.2.0)^[^ ^82^ ^]^ and Silva 138 SSURef NR99 (16S, 18S), Silva 132 LSURef (23S, 28S), FAM v14.1 (5S, 5.8S) database.^[^ ^83^, ^84^ ^]^ To cluster ocean RNA reads, HF reads (deprived of rRNA sequences) were subjected to all versus all alignment Blastn (blastn ‐task blastn ‐db nt ‐query HF_reads ‐out HF_nt_aligned ‐max_target_seqs 1 ‐max_hsps 1 ‐evalue 0.0001) under specific filtering conditions: HF read length of at least 200 bases, alignment length of over 100 bases, and gap openings fewer than 50. The reads were clustered using the Markov Cluster algorithm (MCL; inflation value = 1.5)^[^ ^6^, ^85^ ^]^ based on the network of HF reads sharing > 98% nucleotide sequence identity (**NI**) with > 90% alignment fraction of the shorter read (**AF**). To compare HF reads and short‐read assembled contigs, the same ocean RNA sample (8B) was sequenced with illumina short‐read sequencing and assembled using MegaHit using the same procedures previously described.^[^ ^6^ ^]^ Individual HF reads in each MCL cluster were mapped to MegaHit contig using Blastn (blastn ‐task blastn ‐db nt ‐query HF_reads ‐out HF_nt_aligned ‐max_target_seqs 1 ‐max_hsps 1 ‐evalue 0.0001) with specific filtering criteria: a minimum HF read length of 200 bases, alignment length exceeding 100 bases, and fewer than 50 gap openings.  The match thresholds of 90% NI and 80% AF were applied, which were previously optimized to maximize the distance of “species”‐level virus operational taxonomic units.^[^ ^6^ ^]^ To identify RNA virus sequences from the HF long read data and MegaHit contigs, we performed an approach based on search and update of profile hidden Markov model (HMMs) of virus RdRps (https://doi.org/10.5281/zenodo.5731488) as described previously.^[^ ^6^ ^]^ RdRp domain sequences were extracted using the alignment coordinates between the matching amino acid sequences and HMMs.

### Identification, Phylogenetic Analysis, Annotation, and Host Prediction of Sequences Derived from RNA Viruses

Read sequences were translated with EMBOSS Transeq using 6 frames, replacing the asterisks (stop codons) with Xs, and searched against a hidden Markov model (HMMs) database of the main RNA virus lineages^[^ ^86^ ^]^ and eight new families using the hmmsearch function of HMMER, following Zayed et al. (2022).^[^ ^6^ ^]^ Using the coordinates of the top‐matching alignments between amino acid sequences and HMM profiles, we extracted the portion (footprint) derived from the RdRp domain. The footprints were searched against the nr database with a minimum score of 100 using BLASTP.

We first assessed the corresponding RdRp nucleotide sequences from both the reference and query using 3SEQ.^[^ ^87^ ^]^ A sequence was considered as a recombinant using a Bonferroni corrected *p*‐value cut‐off of 0.05. Though two sequences were identified to be recombinant, they did not meet the *p*‐value cut‐off. The combined RdRp amino acid sequences from the reference and query sequence were subsequently aligned using the E‐INS‐i strategy for 1000 iterations in MAFFT (v 7.017).^[^ ^88^ ^]^ The aligned sequences were trimmed using TrimAl (v 1.2)^[^ ^89^ ^]^ using the ‐gappyout option. Aligned sequences were visualized and manually inspected in Jalview v 2.11.4,^[^ ^90^ ^]^ removing extraneous short sequences, overhangs, and poorly aligned sequences, and detecting six conserved motifs (A, B, C, D, E, and F) within RdRp domains.

To infer the evolutionary relationship among the RdRp sequences, we first determined the appropriate evolutionary models using Model Finder.^[^ ^91^ ^]^ The optimal protein evolutionary model was selected using the Bayesian Information Criterion (BIC) criteria,^[^ ^92^, ^93^ ^]^ and the model LG+F+R4 was found to be the optimal model for our dataset. Phylogenetic trees were inferred using a Maximum Likelihood approach in IQTree (v 2.0).^[^ ^94^ ^]^ Branch support was determined based on 1000 iterations of SH‐like aLRT to obtain bootstrap support values. The bootstrap consensus tree was visualized in iTOL v 6.9,^[^ ^95^ ^]^ with branch support of ≥80% bootstrap shown by brown circles on the phylogenetic tree. The phylogenetic tree was only mid‐point rooted to clarify the relationship sequences. Functional annotation of these RdRp‐encoding sequencing reads was performed by searching against NCBI Conserved Domain Database with e‐value ≤0.01. Virus ORFs were predicted from the nucleotide sequences using NCBI ORF Finder (https://www.ncbi.nlm.nih.gov/orffinder/) with the standard genetic code, using ATG as start codon, excluding ORFs shorter than 300 nts. For short‐read coverage analysis, CoverM was used to calculate short‐read mapping statistics using the bwa‐mem mapper with the following parameters: –min‐read‐percent‐identity 0.95, –min‐read‐aligned‐percent 0.80, –min‐covered‐fraction 0.80, with the default options for the rest of the parameters.

To predict putative hosts of the RNA viruses, we used a previously established approach^[^ ^6^ ^]^ based on the existence of endogenous virus elements, in which RdRp amino acid sequences were searched against genomes of all cellular organisms available in NCBI GenBank (release 243) via tblastn with an e‐value ≤10^−20^.

### Statistical Analysis

All statistical analyses were performed using R (v4.2.2), GraphPad Prism (v10.0), and Microsoft Excel. Welch's *t*‐test was used for pairwise comparisons of continuous variables, including RCA read lengths, subread distributions, and read throughput across experimental conditions. For categorical comparisons—such as the proportion of subreads <1 Kb—two‐tailed *t*‐tests or chi‐squared (χ^2^) tests were applied, as appropriate. Pearson's correlation coefficient was used to assess linear relationships, including the correlation between viral detection frequencies derived from long‐read and short‐read datasets (e.g., Figure 5G). Exact *p*‐values, the number of biological replicates (*n*), and the type of statistical test used are reported in the figure legends and main text. A significance threshold of *p* < 0.05 was used throughout.

### Ethics Approval Statement

SARS‐CoV‐2 sample analysis was based on the approved protocol by The Ohio State University Biomedical Sciences Institutional Review Board (IRB, ID #2021H0080) and by the Ohio State Institutional Biosafety Committee (IBC) (ID #2020R00000046 and #2020R00000030). Each participant provided formal, electronic consent to the following HIPAA AUTHORIZATION TO DISCLOSE PROTECTED HEALTH INFORMATION statement: “I voluntarily authorize OSUWMC to use and/or disclose my COVID‐19 test results to The Ohio State University as part of the ongoing surveillance testing related to COVID‐19 community spread. I understand that my COVID‐19 test results are considered Protected Health Information (PHI) and no payment will be exchanged for disclosure of my test results. I further understand that I have the right to revoke this authorization, in writing, by sending written notification to: Office of Compliance and Integrity‐Privacy, 650 Ackerman Road, Columbus, Ohio 43202. I understand that PHI used or disclosed pursuant to this authorization may be redisclosed by the recipient and its confidentiality may no longer be protected by federal or state law. I consent to the use of an electronic signature and understand that documenting my consent below, I have affirmatively executed this authorization.” Per our IRB‐approved Waiver of Consent Process, we did not seek additional consent beyond that which participants had already agreed (i.e., the above HIPAA AUTHORIZATION TO DISCLOSE PROTECTED HEALTH INFORMATION statement) for the following reasons: 1) Our study used leftover human specimens that were not individually identifiable; 2) the use of each sample posed no additional risk to the original donor than that to which they are already aware (i.e., the potential loss of privacy), and the intent of our study also related to surveillance of COVID community spread, to which donors have already consented per the statement above

## Conflict of Interest

The authors declare no conflict of interest.

## Author Contributions

H.Y. and S.G. contributed equally to this work. S.K., H.Y., S.G., S.F., S.L.L., J.L., M.O., and M.B.S. conceived the conceptualization. S.K., H.Y., S.G., D.X., G.D.H., J.M.W., B.B., S.M., and S.h.K. performed methodology. H.Y., S.G., G.L., S.M., and S.h.K. performed the investigation. S.K., H.Y., S.G., G.L., G.D.H., J.M.W., and B.B. performed formal analysis. S.K., D.X., H.Y., G.L., and S.G. performed the software. S.K., H.Y., and S.G. performed visualization. S.K. and M.B.S. performed supervision. S.K., H.Y., and S.G. performed writing—original draft. G.D.H., J.M.W., S.L.L., J.L., M.O., and M.B.S. performed writing—review & editing.

## Supporting information



Supporting Information

Supplemental Table 1

Supplemental Table 2

Supplemental Table 3

Supplemental Table 4

Supplemental Table 5

Supplemental Table 6

Supplemental Table 7

Supplemental Table 8

Supplemental Table 9

Supplemental Table 10

## Data Availability

All data supporting the findings of this study are publicly available. Nanopore sequencing data are available in the European Nucleotide Archive (ENA) with an accession number PRJEB96931 (ERP179559). The CLAE software toolkit, including code for subread extraction and consensus generation, is freely available on GitHub at: https://github.com/xdtdaniel/CLAE. Any additional materials are available from the corresponding author upon reasonable request.
