# Supplementary material for: CLAE: A High‐Fidelity Nanopore Sequencing Strategy for Read‐Level Viral Variant Detection and Environmental RNA Virus Discovery
Source: Adv Sci (Weinh). 2025 Sep 11;12(44):e05978. doi: 10.1002/advs.202505978 (PMC12667459; doi:10.1002/advs.202505978)
Supplement: Supplementary file 1 — Supporting Information [file ADVS-12-e05978-s005.docx]

Supplementary Materials for

**Enhancing High-Fidelity Nanopore Sequencing for Sensitive Profiling of RNA Viruses**

Hannah Yu *et al.*

*Corresponding author. Email: kim.6477@osu.edu

**This PDF file includes:**

Figures. S1 to S5

Tables. S1 to S10


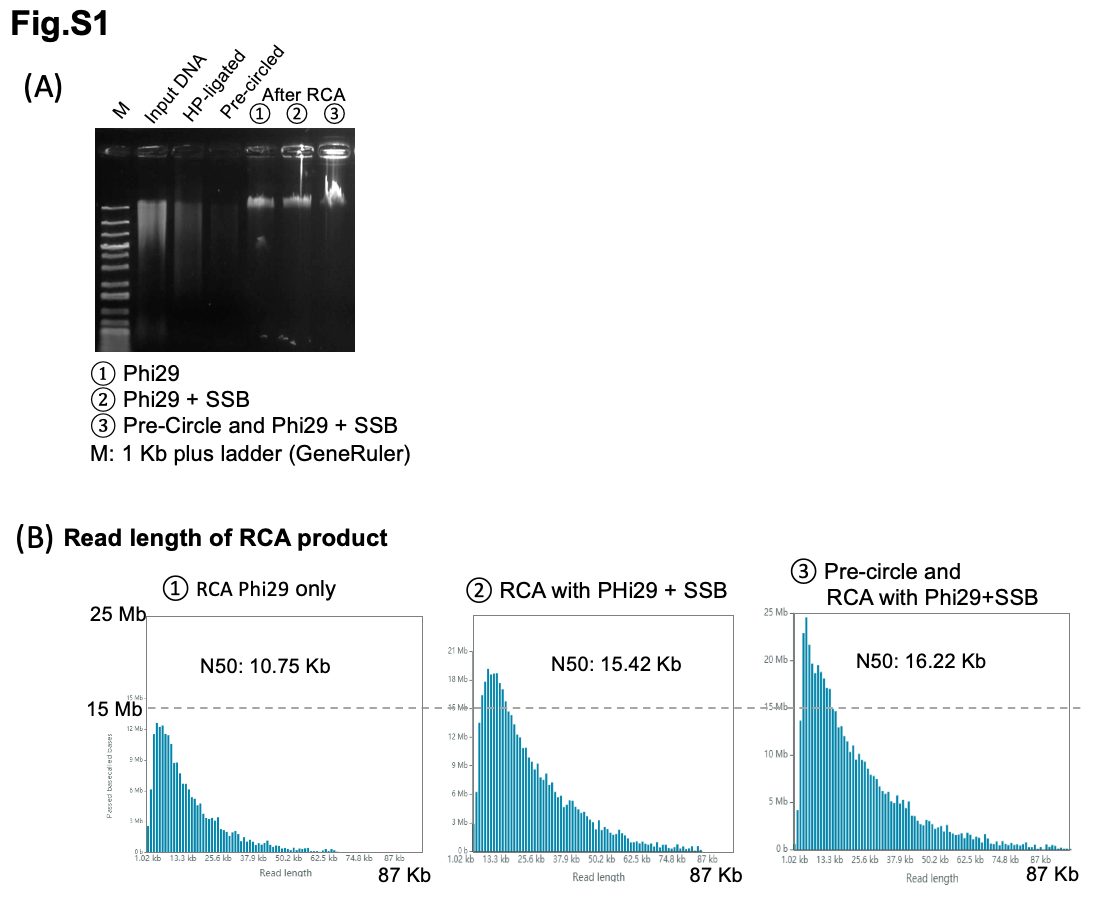


Fig. S1.

**Single-strand binding proteins (SSBs) and a pre-circling step enhance Phi29-mediated RCA. A.** Agarose gel image of input template DNA, hairpin-linker (HP)-ligated template DNA, pre-circled DNA, and RCA-amplified DNA under different conditions: Phi29 only, Phi29+SSB, and pre-circling conditions. 1 Kb plus ladder (GeneRuler) was used (M). **B.** Read length distribution of Nanopore RCA DNA sequencing. Read throughput (y-axis, Mb) for different lengths of RCA DNA reads (x-axis, Kb) are shown for the three RCA conditions, including Phi29 only, Phi29+SSB, and pre-circling.


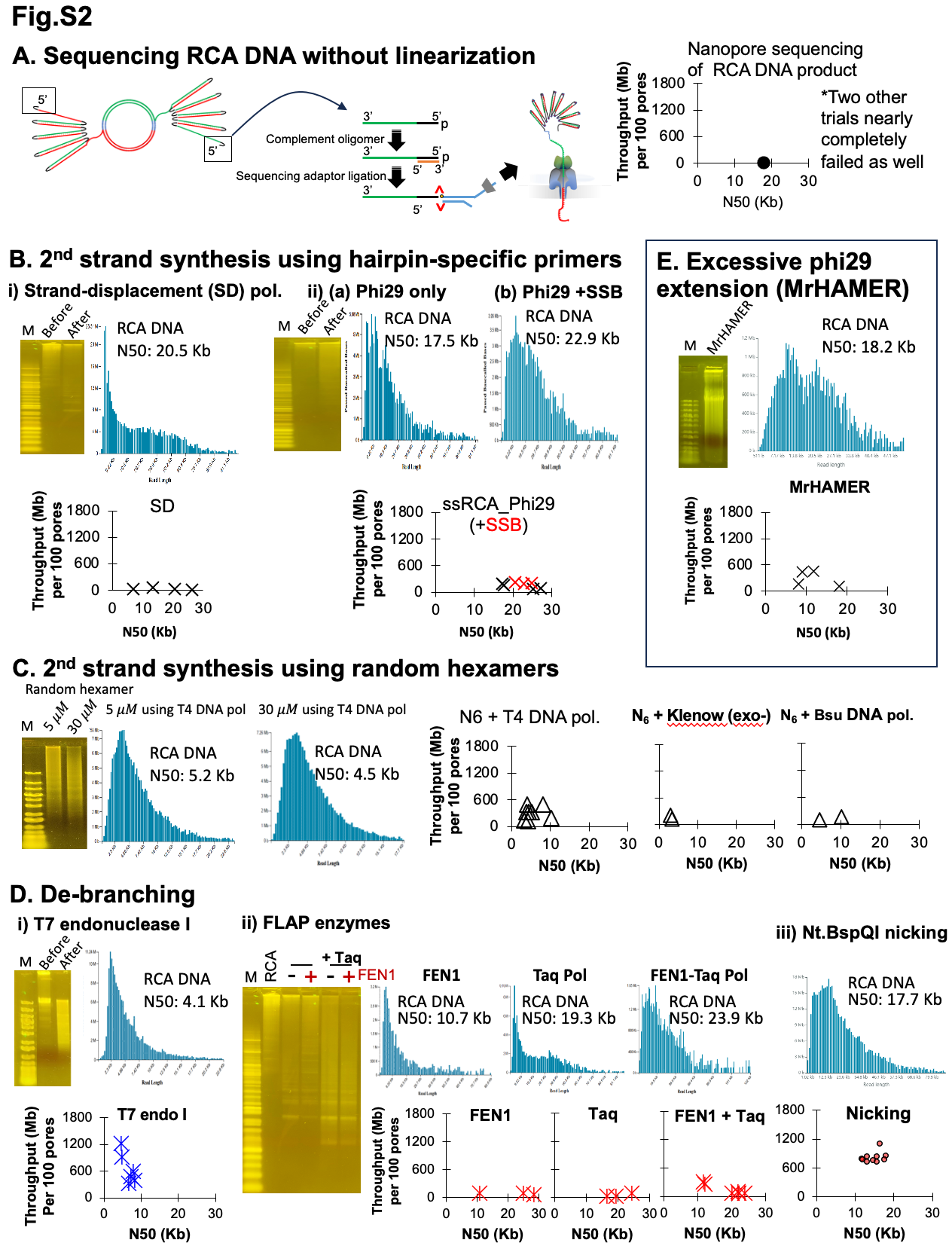


Fig. S2.

**Optimizing linearization and debranching of RCA DNA. A.** Schematic view of Nanopore sequencing of RCA DNA. Oligonucleotides complementary to the 5’ end of RCA DNA (e.g. SecStrand_NtBspQI, SecStrand_NtBbvCI, or SecStrand_NbBbvCI primers) were incubated with purified RCA product and ligated to Nanopore sequencing adaptors according to the manufacturer’s instructions. Sequencing RCA DNA without linearization nearly completely failed. **B-C.** To linearize RCA DNA, complementary (second) strands were generated using hairpin-specific oligos (B) or random hexamers (C). Agarose gel images show RCA DNA before and after the treatment. Read throughput (Mb) per 100 pores (x-axis) and N50 (Kb, x-axis) are shown for each experiment. **D.** For the debranching of linearized (Phi29+SSB) RCA DNA, three methods were tested, including the treatment with T7 endonuclease I (-*i*-), FLAP enzymes (-*ii*-) and nicking enzymes (-*iii*-). **D.** Excessive phi29 extension employed in MrHAMER was tested. The treatment conditions for each of these experiments are available in **Table S2**.


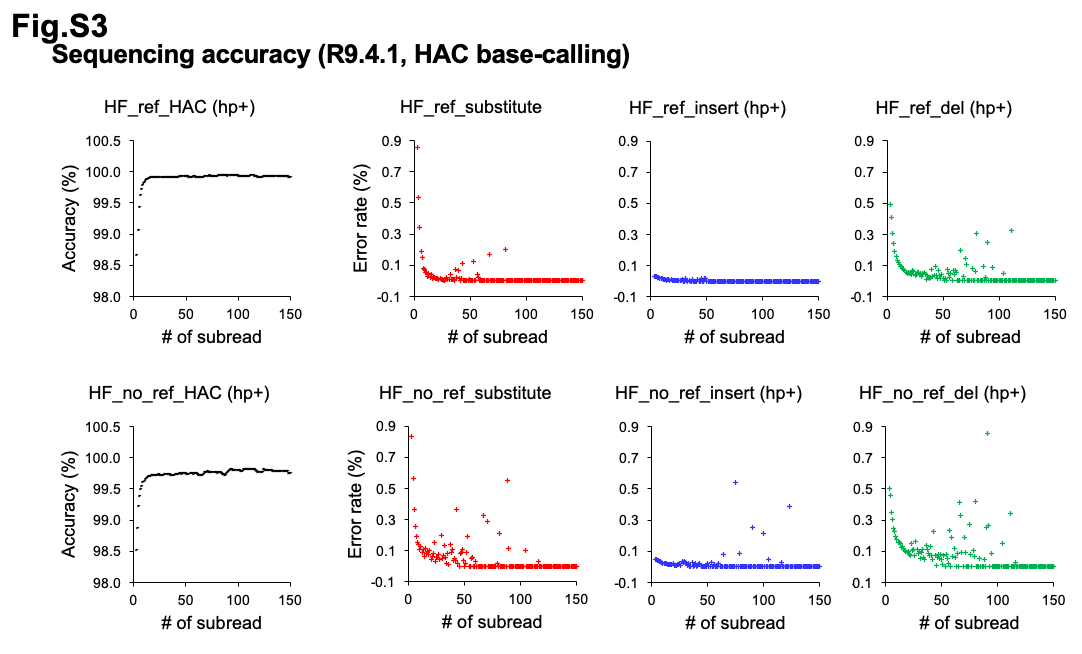
Fig. S3.

**CLAE enables efficient correction of read errors.** Error correction efficiencies of CLAE with a reference (HF_ref) and without a reference (HF_no_ref) were compared. Read errors were more effectively corrected for RCA reads that have higher number of subreads. Nevertheless, correcting homopolymer errors (hp+) remained challenging even using more than 50 subreads.


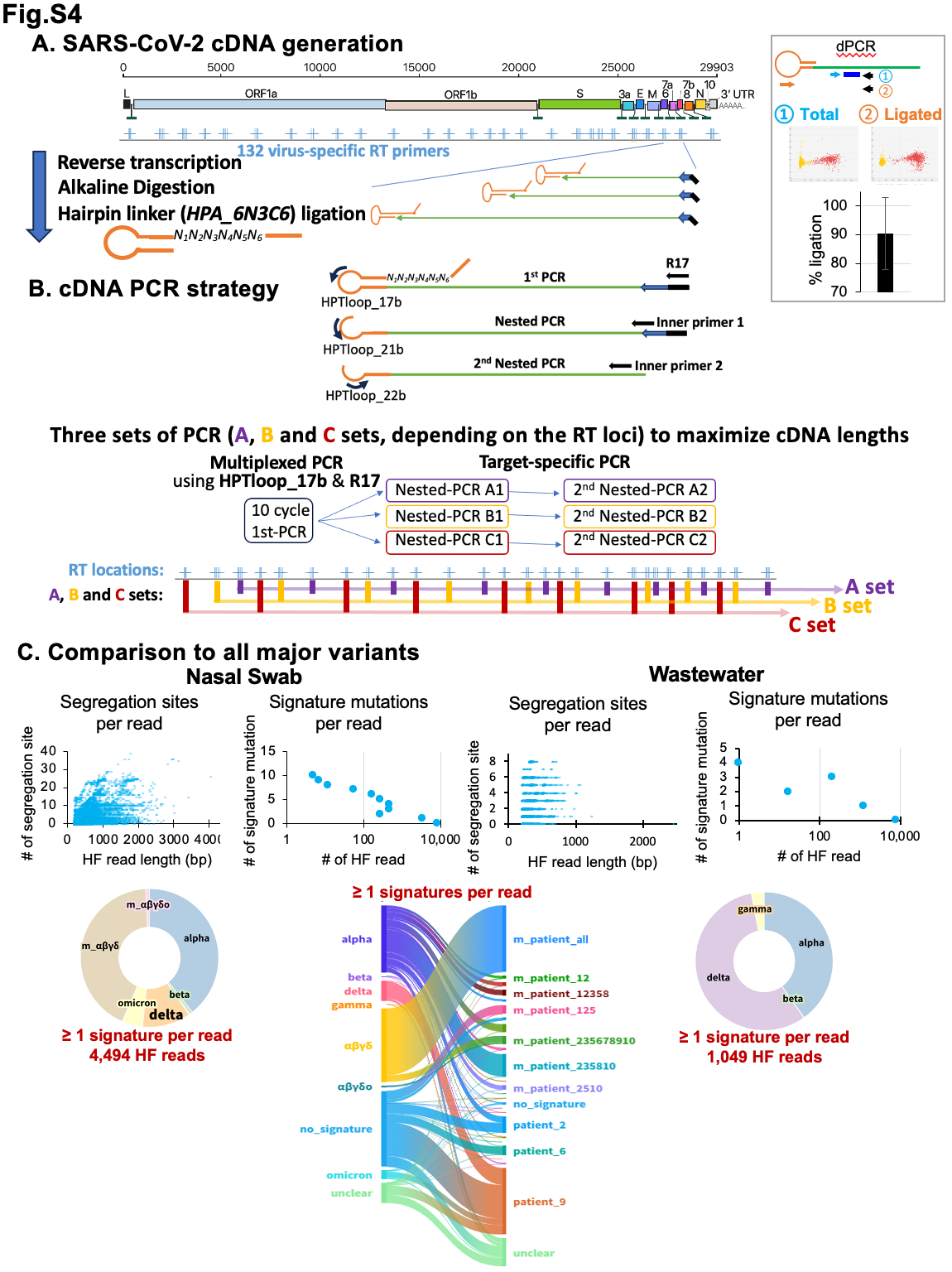


**Fig. S4. CLAE enables read-level detection of local SARS-Cov-2 variants.** A-B. Schematic view of SARS-CoV-2 sequencing procedures. SARS-CoV-2 RNAs were reverse-transcribed using 132 virus-specific RT primers (blue crosses). Viral cDNAs were then ligated to HPA_6N3C6 (hairpin) linkers. The hairpin ligation efficiency was measured by digital PCR (dPCR) (boxed area). Linker-ligated cDNAs were than subjected to a three-step PCR designed to maximize cDNA lengths and minimize PCR artifacts. Briefly, the first PCR was carried out using HPTloop_17b and R17 primers common in all cDNA (10 cycle only). Then the amplified DNAs were purified and divided into 3 groups (A, B, and C), each with unique sets of primers that amplify cDNAs of specific locations (purple bars for A set; orange bars for B set; and red bars for C set). Primer sets for A, B, C PCR sets can be found in Table S6. C. Nasal swab and wastewater HF reads were compared to the reference mutations of global major SARS-CoV-2 variants (see Table S9 for VOC types and signature mutations).

**
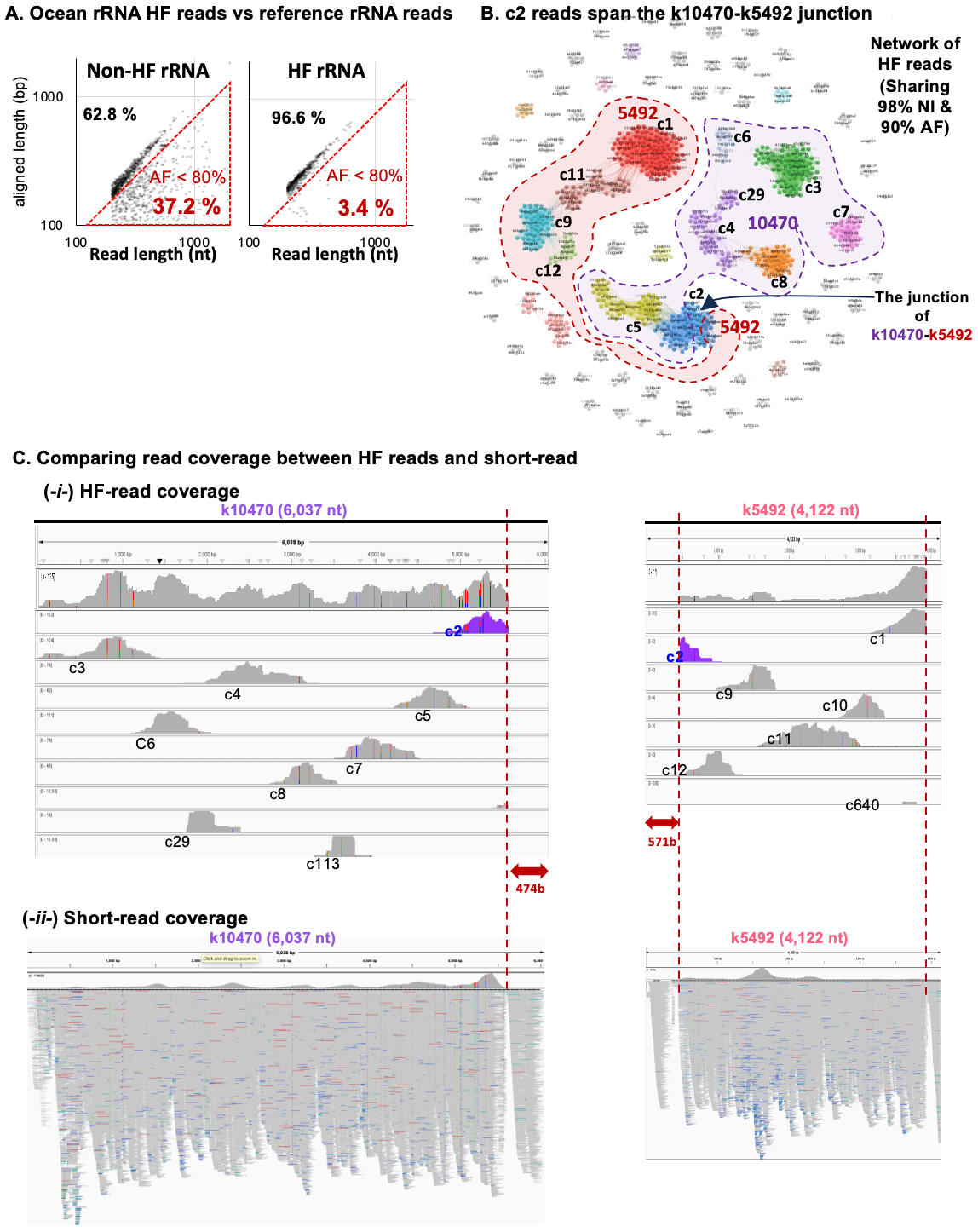
**

**
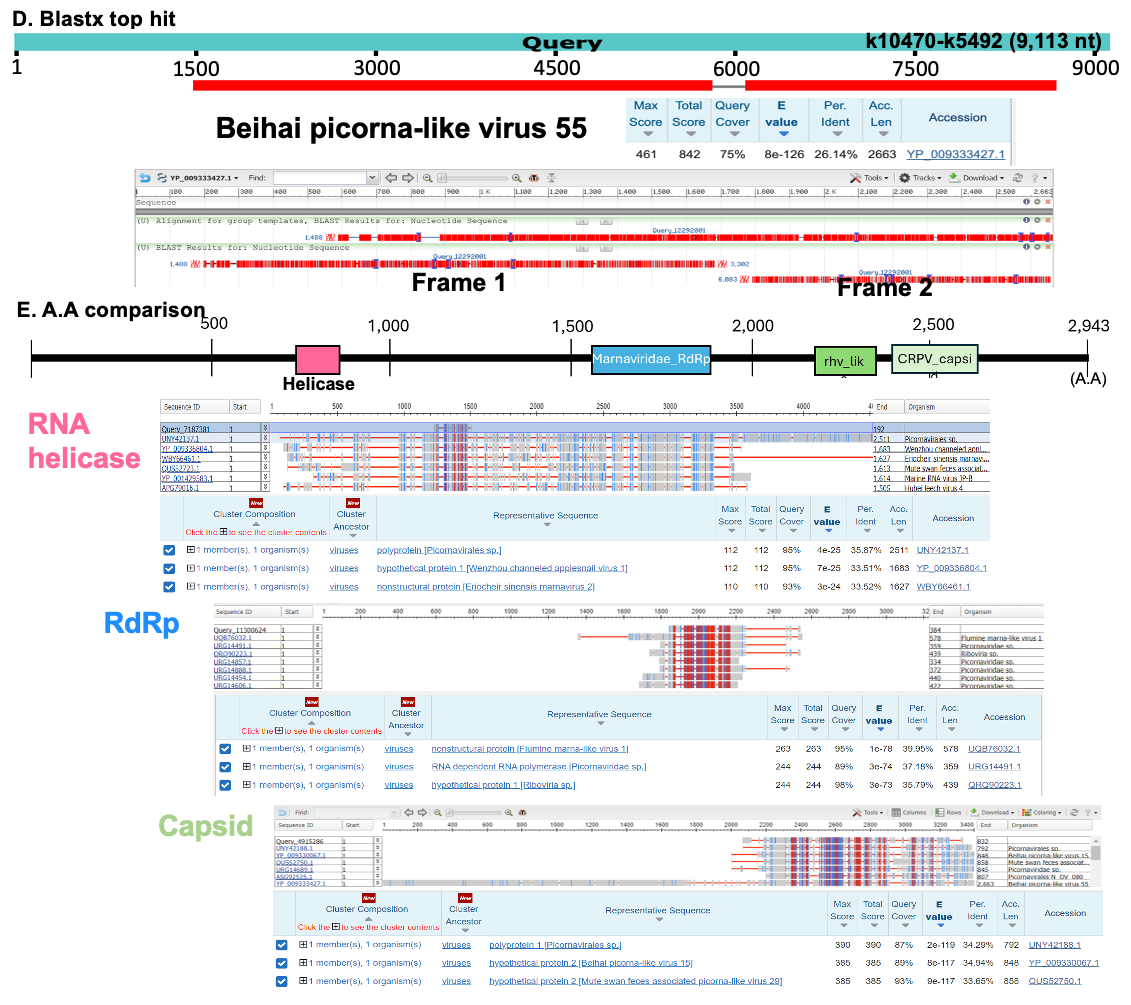
**

**Fig. S5. Agnostic long-read HF sequencing reveals novel RNA viruses.** **A.** Comparison of ocean rRNA reads with the eukaryotic rRNA reference sequences. HF reads with AF < 80% are shown within the red triangle. **B.** Network of HF reads sharing 98% NI and 90% AF (VOSviewer). HF reads were clustered using MCL. Clusters that map to k5492 (red area) and k10470 contigs (purple area) are shown. c2 group show alignment to both clusters. Part of c2 reads also align to the junction of the k10740-k5492 fusion contig. **C.** Comparing the read coverage between HF-reads and short-read data. The potential mis-assembly sites (read dotted lines) show substantially lower read coverage than other regions. **D.** The k10470-k5492 contig is closely related to “Beihai picorna-like virus 55” with 75% genome (e-value of 8e-126 by Blastx), potentially containing two separable open reading frames: one encoding helicase and RdRp and the other encoding capsid. **D.** Amino acid sequences of the helicase, RdRp, and capsid showed 35.9%, 40.0% and 34.3% similarity, respectively, to those of picornavirads (95% coverage with e-value of 4e-25), marnavirads (95% coverage with e-value of 1e-78), and picornavirads (87% coverage with e-value of of 2e-119).

Table S1.

*Attached as separate file

Summary of current high-fidelity (HF) Nanopore sequencing methods. The table shows key technical components of current HF methods that affect read-length bias and read throughput, including Pre-RCA sample preparation, linearization and debranching methods.

Table S2.

*Attached as separate file

Linearization and Debranching Efficiency. The efficiency of tested conditions concerning the linearization and debranching steps while optimizing sequencing of RCA DNA. It includes reaction conditions: enzyme tested, template DNA and primers used, and experimental conditions. The outcome of each trial is presented as N50 (kb) and throughput (Mb/100 pores) following the sequencing of each condition. Of the linearization testing conditions, strand extension mediated by Phi29 in the presence of SSB (Phi29+SSB) enhanced read length, achieving an N50 of at least 23 Kb. Debranching with target-specific nicking endonuclease Nt.BspQI showed consistent improvement of read-throughput.

Table S3.

*Attached as separate file

HF Read Recovery Rates for R9.4.1 and R10.4.1 flow cells. When an equal number of subreads was analyzed, SUP mode outperformed HAC and FAST modes in error correction for both R9.4.1 and R10.4.1 data, achieving 9.9% to 12.8% more >Q30 reads and 7.7% to 9.8% more >Q40 reads than HAC mode.

Table S4.

*Attached as separate file

SARS-CoV-2 Nasal Swab Short-Read Sequencing Report. Lineage, status of sequencing quality, note, % of non-N bases, median coverage, and IDI sample # assigned to each of 10 nasal swab samples obtained and Illumina sequenced by OSU IDI.

Table S5.

*Attached as separate file

SARS-CoV-2 Nasal Swab Short-Read Mutation Profiles. 10 patient SARS-CoV-2 mutation profiles with their individual IDs, clade identification, and mutation sites defined by Illumina sequencing.

Table S6.

*Attached as separate file

Oligos. Primer name and sequences used for SARS-CoV-2 reverse transcription, PCR, cDNA hairpin ligation, RCA hairpin ligation, and subsequent sequencing and linearization primers. Primers for digital PCR verification are also included. All primers were ordered through IDT.

Table S7.

*Attached as separate file

Global Alpha Variant Reference. Signature mutations present in four SARS-CoV-2 Alpha variants with their prevalence. Global VOC reference was downloaded from the “lineage reports” section of COVID-19 CG ([covidcg.org](https://covidcg.org/))(*72*) with options of “Mutation Type: NT” and “Consensus Threshold : 0.7”.

Table S8.

*Attached as separate file

Nasal Swab Reference. 128 segregation sites present in our local nasal-swab Illumina sequencing data with the prevalence of each signature mutation present in each nasal-swab sample.

Table S9.

*Attached as separate file

Global Major Variants. Signature mutations present in 21 SARS-CoV-2 variants with their prevalence. Global VOC reference was downloaded from the “lineage reports” section of COVID-19 CG ([covidcg.org](https://covidcg.org/))(*72*) with options of “Mutation Type: NT” and “Consensus Threshold : 0.7”.

Table S10.

*Attached as separate file

Sequencing Data Summary. Sequencing data locations for source data within each figure presented in the main text. These files will be uploaded to the European Nucleotide Archive.
